# Fluorescence based rapid optical volume screening system (OVSS) for interrogating multicellular organisms

**DOI:** 10.1038/s41598-021-86951-3

**Published:** 2021-04-07

**Authors:** Jigmi Basumatary, Tarannum Ara, Amartya Mukherjee, Debanjan Dutta, Upendra Nongthomba, Partha Pratim Mondal

**Affiliations:** 1 Nanobioimaging Lab., Department of Instrumentation and Applied Physics, Indian Institute of Science, Bangalore, 560012 India; 2 Department of Molecular Reproduction, Development and Genetics, Indian Institute of Science, Bangalore, 560012 India

**Keywords:** Biophysics, Health care, Medical research, Optics and photonics

## Abstract

Continuous monitoring of large specimens for long durations requires fast volume imaging. This is essential for understanding the processes occurring during the developmental stages of multicellular organisms. One of the key obstacles of fluorescence based prolonged monitoring and data collection is photobleaching. To capture the biological processes and simultaneously overcome the effect of bleaching, we developed single- and multi-color lightsheet based OVSS imaging technique that enables rapid screening of multiple tissues in an organism. Our approach based on OVSS imaging employs quantized step rotation of the specimen to record 2D angular data that reduces data acquisition time when compared to the existing light sheet imaging system (SPIM). A co-planar multicolor light sheet PSF is introduced to illuminate the tissues labelled with spectrally-separated fluorescent probes. The detection is carried out using a dual-channel sub-system that can simultaneously record spectrally separate volume stacks of the target organ. Arduino-based control systems were employed to automatize and control the volume data acquisition process. To illustrate the advantages of our approach, we have noninvasively imaged the *Drosophila* larvae and Zebrafish embryo. Dynamic studies of multiple organs (muscle and yolk-sac) in Zebrafish for a prolonged duration (5 days) were carried out to understand muscle structuring (Dystrophin, microfibers), primitive Macrophages (in yolk-sac) and inter-dependent lipid and protein-based metabolism. The volume-based study, intensity line-plots and inter-dependence ratio analysis allowed us to understand the transition from lipid-based metabolism to protein-based metabolism during early development (Pharyngula period with a critical transition time, $\tau _c = 50$ h post-fertilization) in Zebrafish. The advantage of multicolor lightsheet illumination, fast volume scanning, simultaneous visualization of multiple organs and an order-less photobleaching makes OVSS imaging the system of choice for rapid monitoring and real-time assessment of macroscopic biological organisms with microscopic resolution.

## Introduction

The field of developmental biology routinely uses model organisms (such as Zebrafish and *Drosophila*) to understand organ growth and regeneration beginning from embryonic to adult period. Such studies in Zebrafish inform us about the onset of the formation of the dorsal-ventral stripes, pectoral fins and the circulatory system (including, the heart). In addition, these organisms have aided our understanding of cardiovascular and neuronal diseases and their progression^1–3^. A major advance in quantifying these studies requires a non-invasive optical fluorescence microscope with rapid volume imaging capability, large field-of-view, diffraction-limited resolution and minimal photobleaching. Compared to conventional point-scanning technique, the light sheet based plane-scanning technique is faster and has the advantage of minimal photobleaching and phototoxicity^4–6^. In addition, such microscopy technique may facilitate inter-organ communication studies and prolonged study of the entire multicellular organism in its natural physiological state.

The light-sheet-based microscopy is quickly gaining importance as the scope-of-choice due to its large field-of-view (FOV) imaging, high contrast (consequence of orthogonal detection geometry) and diffraction-limited resolution^7–11^. Over the last few years, many important variants of light-sheet fluorescence microscopy (LSFM) have emerged. Some of these include thin light-sheet microscopy^12^, ultramicroscopy^13^, objective coupled planar illumination microscopy (OCPI)^14^, confocal light-sheet microscopy^15,16^, multiple light-sheet microscopy^17^, dual-inverted selective-plane illumination microscopy (diSPIM)^18^, light-sheet theta microscopy (LSTM)^19^, open-top light-sheet (OTLS)^20,21^, coded light-sheet array microscopy (CLAM)^22^ and lattice light sheet microscopy^23^. The advantages offered by LSFM and its variants have seen an upsurge in the studies related to large biological specimens^9,24–26^. Recently few groups (Keller, Fraser and Huiskin) have shown impressive time-series of entire embryo^27–31^. However, the standard protocol requires visualization of a specific specimen plane at a time and thus suffers from missing information from other sections of the whole specimen. The large data acquisition required in traditional light-sheet microscope (LSM) slows-down the volume reconstruction process. LSM techniques require both translation and rotation of specimens, whereas proposed OVSS employs only rotation for data collection. This substantially reduces data acquisition time and thereby fastens volume imaging. So there is a need to accelerate light-sheet based volume imaging and screening system with the capability of simultaneous multichannel imaging and long-time monitoring. This will ensure real-time volume interrogation of drug-receptor interaction and fluorescence label-based assays^11,32^. An advanced version of such a system would be able to track and locate target proteins complexes and drug molecule clusters in the organs during early development.

Fast imaging using light sheet is demonstrated in a variety of techniques. Truong et al, showed faster imaging using light sheet when compared to point scanning techniques^33^. In another application, a high-speed volumetric calcium imaging was demonstrated by Prevedel et al using light-field microscopy^34^. A fast version of light sheet microscopy called SPED was recently demonstrated that show fast sub-cellular resolution imaging of clarity mouse brains and volumetric Ca(2+) imaging of entire zebrafish nervous systems^35^. Recently, Greer et al. has presented a new speed-optimized Objective Coupled Planar Illumination (OCPI) microscopy for fast calcium imaging of the larval zebrafish brain^36^.

To overcome the limitations (such as, prolonged data acquisition, slow volume reconstruction, small FOV and severe photobleaching) of existing light-sheet microscopy, it is necessary to develop a rapid multicolor volume imaging system. Existing LSM systems require light-sheets to be scanned throughout the specimen in a translation-rotation configuration, but this process demands large scanning time and big data-storage device when compared to rotation-based OVSS system. It is thus imperative to design advanced light-sheet microscopes that are simple in geometry and readily adaptable to the existing systems. A simple yet efficient route to address these challenges is to eliminate translational scanning altogether and rely solely on rotational scanning integrated with reconstruction techniques for volume imaging. Rotation ensures isotropic data collection as compared to linear scanning employed in traditional LSM and its variants.

Rotation-based isotropic scanning is attractive as this boosts-up the speed by many folds. This comes with the fact that OVSS requires interpolation to determine data on the rectangular-grid (see, Supplementary 3). As compared to linear interpolation that is extensively employed in LSM^36–38^, we have used cubic interpolation which is more precise. However, note that, the image treatment (interpolation) can improve the visual appearance but it cannot recover missing information. So, the real-time advantage comes with a balance between the speed (data acquisition time) and information content. Using sampling theory, the ideal sampling is found to be approximately 3 degrees for 7.5 micron thick light sheet. Hence 60 2D-angular image are enough to reconstruct volume-stack. From the data acquisition timing diagram, it is found that OVSS method takes 42 ms/frame and a total of 60 images whereas, traditional LSFM takes a much larger number of images for volume reconstruction. In general, OVSS is faster than traditional SPIM. Moreover, it was found that high rotation speed of stepper motor results in mechanical errors due to the vibration.

Here, we demonstrate advanced OVSS imaging system that provide rapid screening and real-time volume visualization of large specimens with the added advantage of multi-organ interrogation. Alternately, the technique is also called Lightsheet Illuminated Volume Expedite (LIVE) Microscopy. To demonstrate the versatility of our OVSS system, we performed studies on muscle development and lipid accumulation in the Zebrafish from pharyngula period to larval period. Our OVSS system facilitates the study of multiple organs and abnormalities during development, aiding non-invasive probing with great potential in health-care and therapy.

## Results

The prolonged data acquisition time and severe photobleaching prohibits real-time visualization of rapidly-occurring biological processes during early development and so there is a necessity to develop fast volume imaging system that can image an entire organism with minimal optical artifacts and are free from photobleaching effect. During preliminary investigation, we observed that organs labeled with fluorophores (Yolksac stained with BODIPY) result in faster bleaching due to short bleach-time constant. So the saturation is avoided during data acquisition. It is however important to note that photobleaching remains a serious concern for rapid volume imaging^30,39^. Detailed photobleaching characteristics of the fluorophores (BODIPY, DsRed and TRITC) used in the present biological study can be found in Supplementary 4.

### Fast scanning and detection

**Figure 1:**
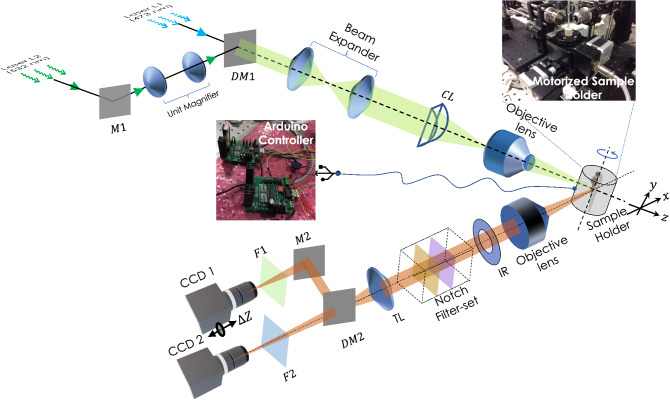
Schematic diagram of multicolor Lightsheet based OVSS fluorescence system. Two distinct wavelength of light sources ($\lambda _1 =473$ nm and $\lambda _2 =532$ nm) are used to excite spectrally-distinct fluorophores. A unit magnifier (with lens separated by a distance $> (f_1 +f_2)$) is placed in one of the illumination arms to correct for the focal-shift ($\Delta Z$) due to chromatic aberration induced by 10$\times$ objective lens. Beams are combined by Dichroic mirror (DM1), expanded and passed through cylindrical lens (CL, $f=150$ mm) to generate the light sheet. Additionally, we placed an objective lens (Olympus, 10$\times$, 0.3 NA) at the focus of cylindrical lens to generate diffraction-limited multicolor lightsheet (MLS). The specimen (encaged in a capillary tube) placed on the automated rotating-stage is illuminated by MLS. To observe the fluorescence, an orthogonal detection system is employed that use a separate detection objective lens (Olympus, 4X, 0.1 NA/Olympus, 10$\times$, 0.25 NA) to collect the image data. Iris (IR) and Notch filters (473 NF and 532 NF) are used to cut-off the nonparallel and scattered light respectively. Bandpass fluorescent filters (range $500\pm 12$ nm) along with the dichroic mirror DM2 (with cut-off, $\lambda _c = 505$ nm), additional filters (F1 and F2), mirror (M2) and tube-lens (TL) are used to divert, filter and focus beam to the respective cameras ports (CCD1 and CCD2) for data recording.

The proposed OVSS imaging has a simple geometry that enables simultaneous multi-organ volume imaging and long-time interrogation. Few new features are introduced in LSM microscopy that includes, multicolor lightsheet illumination, rotation-based angular 2D data collection and simultaneous multicolor detection. This led to faster multichannel data acquisition for volume reconstruction (see, Fig. 1). The illumination sub-system is designed to generate diffraction-limited multicolor (combined blue and green) lightsheet point spread function (PSF). A unit magnifier is used to match the focus of lightsheets. The specimen is encaged inside a capillary tube filled with Agarose gel-matrix and the angular motion is controlled using Arduino-controller board (see, Supplementary 1). The multicolor fluorescence thus obtained is collected by detection objective, filtered by the notch-filter set and focussed on to the camera chip. A dichroic mirror (cut-off, $\lambda _c =505$ nm) is used to split the emission fluorescence before being directed to the CCD detectors. Double-arm detection is employed to ensure simultaneous data acquisition of large specimen labelled with multiple fluorophores. This approach allows 3D image stacks to be acquired simultaneously for both channels and this becomes the key for multicolor volume imaging of live specimens. Dedicated reconstruction techniques (including, interpolation and coordinate-transformation) are developed for volume reconstruction from the recorded 2D angular data.

As compared to state-of-the-art light sheet microscopy (LSM) imaging, OVSS provides an alternative system for rapid multicolor scanning, monitoring and whole volume visualization. As an example, light-sheet techniques (MuVI-SPIM) require $>4000$ images for volume reconstruction^40^. This calls for massive memory system and faster computing engines for volume reconstruction. Real-time volume imaging is simply unthinkable for such a system. In general, a single dataset occupies $>1$ GB memory space and so there is a great need for lightsheet techniques that are rapid, portable and require minimal computational resources (less memory and standard processors). With an exposure of 10 ms, motor repositioning time of 22 ms and 60 angular image data, OVSS requires a memory of $\approx ~30$ MB. Thus OVSS imaging enables developmental studies of large specimens in near real-time. Here, we choose to work with 60 angular image data (rotation sampling angle $=3^\circ$). This is primary based on the Shannon’s entropy (information content) ( Supplementary 7).

### Characterizing lightsheets

**Figure 2:**
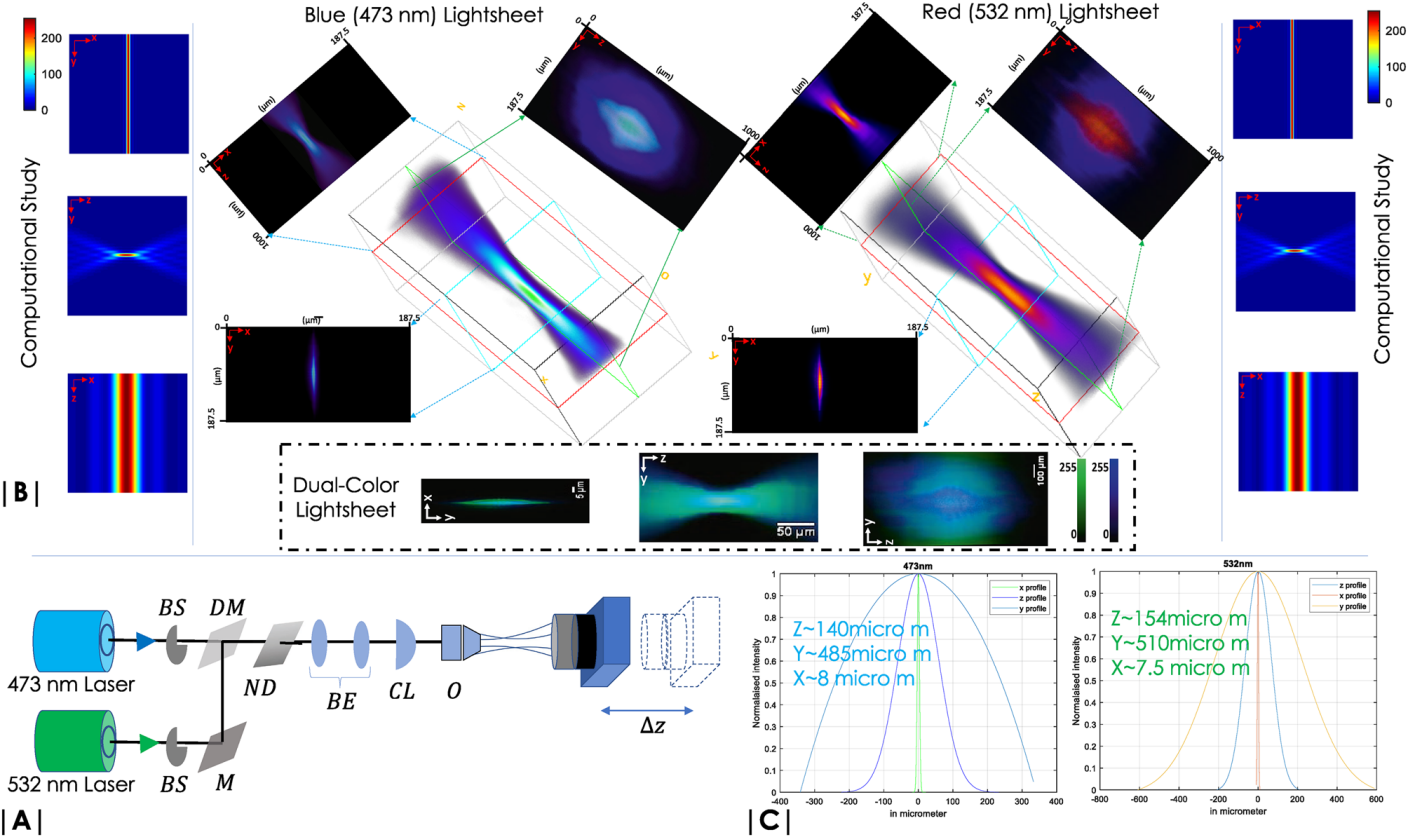
Multicolor lightsheet characterization: (**A**) Schematic diagram of the optical set-up used for light sheet field measurement. Beam shutters (BS) along with mirror (M), neutral density (ND) filters are respectively used to select and scale light intensity in each arm. The beams are combined using a dichroic mirror (DM), expanded 4-times by beam-expander (BE) to completely fill the back-aperture of cylindrical lens (CL). An objective (O) is placed at the focus of cylindrical lens to generate diffraction-limited multicolor light sheet. (**B**) The field is directly recorded by selecting the desired light (473 nm/532 nm) using BS and a CCD camera which is scanned ($\Delta Z = 10\; \upmu$m) about the focus of the objective to record the illumination field intensity. The corresponding 3D and 2D views of the field are shown. The recorded fields are also compared with the computational study. Finally, a dual-color light sheet is also shown. (**C**) Line intensity plots of the field along *X*,  *Y*,  *Z* are used to determine the actual dimension of the light sheet.

The quality of images acquired is strongly dependent on the electric field distribution at the focus and in the neighborhood. A well-focused lightsheet reduce the plane thickness and thereby greatly improves signal-to-background ratio. Two approaches were adopted: (1) Computationally determining the light-field from the first principle^41^, and (2) Direct measurement of light-field by translating a camera in the beam-path^42,43^. First technique requires incorporation of key experimental parameters (such as, wavelength, aperture angle/numerical aperture (NA) and refractive index of objective immersion liquid) as variables in the computational study. Using vectorial theory of light^41,44^, the field was calculated at each point within a grid-size of $256 \times 256$ in the XY-plane. In the field-calculation, we have used an incident light of wavelengths, $\lambda _1 = 473$ nm, $\lambda _2 = 532$ nm, and focussing objective lens of aperture angle, $\alpha =30^\circ$ and a focal-length $f=10$ mm. The resultant field distribution is shown in Fig. 2B (left- and right-sides).

The second approach requires generation of multicolor lightsheet for simultaneous monitoring of multiple organs for a prolonged period of time. Figure 2 shows the field structure at and around the focus for both blue light, ($\lambda =473$ nm) and green light ($\lambda =532$ nm) illumination. A CCD camera was directly placed in the beam-path and z-scanned to record the field at each *z*-increment (see, Fig. 2A). Beam-shutters (BS) were introduced in the beam-line to selectively scan the field. Step Gradient neutral density (ND) filters were introduced to avoid saturation of intensity at the camera. The 3D recorded field along with its XY, YZ and XZ views are shown in Fig. 2B. Strong confinement of the field is evident along the z-axis resembling sheet-like field structure for both the illuminations. An overlapping dual-color light sheet is also shown. Figure 2C shows the intensity line plots across the X, Y, Z axes of the field.

A multicolor lightsheet PSF is desired for multicolor OVSS imaging system. This necessitates complete overlap of the individual single color fields (473 nm and 532 nm). Figure 2B show overlapped PSF that has a thickness of $7.5 ~\upmu$m, height of $485~\upmu$m and a z-extent of $140~\upmu$m. The multicolor PSF is used to monitor and simultaneously image multiple organs during early development stages of Drosophila and Zebrafish.

### Test sample: green and red fluorescent microspheres in agarose-gel matrix

**Figure 3:**
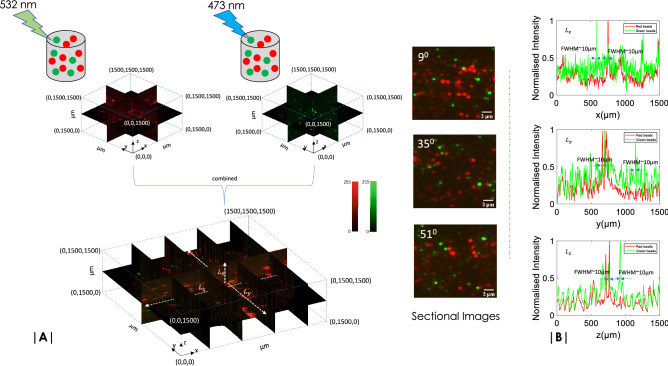
(**A**) OVSS system is calibrated with standard fluorescent beads, Nile Red (Ex/Em: 535 nm/570 nm, ) and Yellow Green (Ex/Em: 505 nm/515 nm). Images are captured at 3 degree rotation with an exposure time of 10 ms and an internal camera gain of 15 dB. A image size of 400 $\times$ 400 pixel size is cropped (see Supplementary 3) and processed. Reconstructed volume slices of different planes along with few sectional images (at $\theta = 9^\circ , ~35^\circ , ~51^\circ$ ) are also shown. (**B**) Line plots (along dotted white lines) of slice at different cross-sections of the volume indicate red and green beads that have a FWHM size of $\approx 10 \; \upmu$m.

As a first step towards multicolor OVSS imaging it is essential to calibrate the system and validate it with a known sample. We have used a random mix of 473 nm and 532 nm excitable fluorescent micro-spheres for which the emission spectra peaks at 515 nm (Green window) and 575 nm (Red window), respectively. The beads were embedded in the Agarose gel-sample as per the developed protocols described in the Methods section. Figure 3A shows 3D reconstructed volume of the sample that is simultaneously exposed to 473 nm and 532 nm light. Using a dichroic mirror (with a cutoff wavelength of 515 nm), the imaging system (see, Fig. 1) is able to simultaneously record angular (yz) plane image data in each detection arm (designated as Green and Blue channel). We observed minimal cross-talks which suggests negligible bleed-through. The recorded data were subjected to 3D volume reconstruction algorithms and subsequently merged to obtain multicolor reconstructed volume stack.

To access the quality of reconstruction, we have used intensity line plots across X, Y and Z axes. Figure 3B shows the corresponding plots for white dotted lines ($L_x, ~L_y, ~L_z$) across the reconstructed volume (shown in Fig. 3A). The plots suggest that the average size for both Green and Red beads [(Invitrogen (F8819, F8823)] are approximately 10 microns. This value is close to the magnified size of the bead, which is approximately 8 microns (1 micron sized bead multiplied by total 8$\times$ magnification of the detection sub-system). The intensity plots roughly determines the lateral resolution of OVSS imaging system to be $\approx 10 ~\upmu$m. With an orthogonal detection system in place, the background is strongly suppressed and technically, contrast is better. We observed a contrast improvement of $\approx 1.425$ times as compared to widefield system. With a widefield resolution of detection sub-system and high contrast, we expect to see the internal structure of organs and their development in early stages of the Zebrafish embryo and *Drosophila* larvae (see, Supplementary 8).

### Time-lapse imaging (double transgenic line)

**Figure 4:**
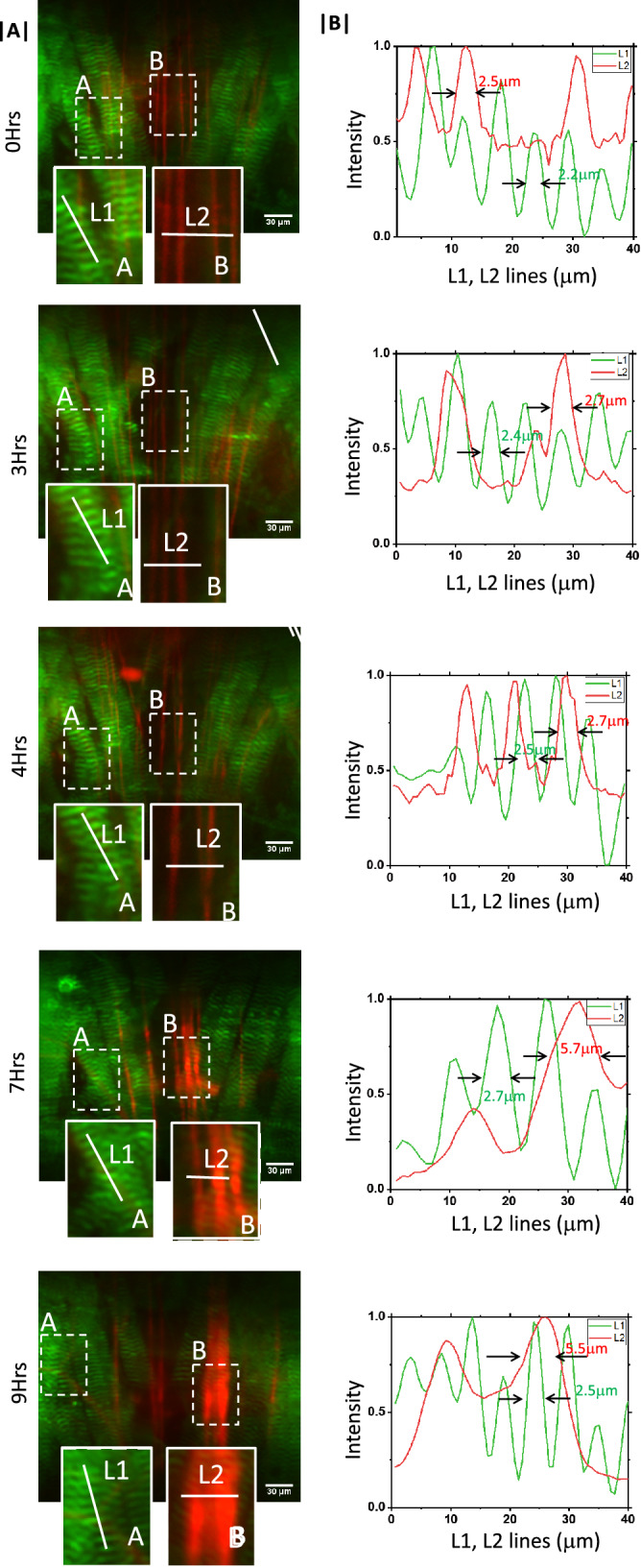
(**A**) 10 h time-lapse imaging of transgenic Drosophila sample showing the development of muscle and neuron over time. (**B**) The corresponding intensity plots that demonstrates the periodic structure of muscle and its development over time. Visualization of fine periodic structures are made possible by high resolution sCMOS camera.

In-vivo time-lapse imaging experiment (e.g. double transgenic line) is performed. Figure 4 shows continuous monitoring of specimen (Drosophila) for a long 12 h study. Volume images are collected after every 2 min for both the channels with an exposure of 30 ms. Recorded 3D volume images in individual channels, and related details are discussed in Supplementary 9. In addition, the acquired complete time-lapse raw-data can be found in Supplementary Video 3. We have used a high resolution sCMOS camera (Andor Zyla 4.2, quantum efficiency of $84\%$) to collect the volume-stacks.

Drosophila larvae are in a continual state of crawling, foraging for food to meet the demand of a nutrient-dependent critical weight in order to undergo metamorphosis^45^. Natural crawling behaviours include turns, head sweeps, pauses, hunching, bending, burrowing, rolling (escape) and forward and backward locomotion^46^. Such a wide repertoire of behaviours requires coordinated interactions between the motoneuron and the muscle. During Drosophila development, the motor neurons and muscles differentiate synchronously, offering an excellent model system for studying intercellular communication between the two cell-types during synapse formation^47^. We examined the existence of such interactions by interrogating the thoracic region of the third larval instar. Hence, the larval somatic body wall muscles and the motor neurons that innervate them are chosen for the present study. Muscles were visualised using the localisation pattern of Zasp52, a core component of the Z-disc in Drosophila muscles, which are organising centres that establish and maintain myofibril structure and function. On the other hand, we visualised neurons by driving the expression of the reporter construct CD8-mRFP throughout the nervous system using nSyb-Gal4.

Figure 4A show a specific section of the specimen where both muscle and neurons can be seen. A sample raw volume data is shown in Supplementary Video 4 as a movie. High resolution images show the presence of periodic pattern in the muscle and neurons with a width of $2.5\;\upmu$m and $2.2\;\upmu$m at 0 h. This pattern changes over time as can be seen from images and intensity plots at 3–10 h. Specifically images recorded at 7 h and 9 h show broadening of neuron structures with a size that is double when compared to neurons at 0 h. The high resolution images clearly demonstrates the capability of OVSS light sheet system for time-lapse imaging.

### Imaging Zebrafish embryo

The parallel study of multiple organs and their interdependence during development is not well understood. A little is known about the metabolic cycle as the development of multiple organs take place in the early stages of organism development. We follow development of organs and even entire organisms in detail, beginning from early pharyngula stage to larval stage of the Zebrafish using OVSS, which is a non-invasive imaging technique to study the development of tissues in Zebrafish. This technique requires separate fluorescent labeling of two different organs (muscle and yolk-sac) with specific fluorescent probes. We have stained muscle using phalloidin-TRITC and yolk with BODIPY. Phalloidin is a bicyclic toxin peptide, commonly used to identify filamentous actin (also known as F-actin) present in the muscle Dystrophin whereas BODIPY is a fluorescent dye used to stain the lipid-rich yolk-sac (primitive Macrophages) (see Methods section for specific protocols)^48^.

**Figure 5:**
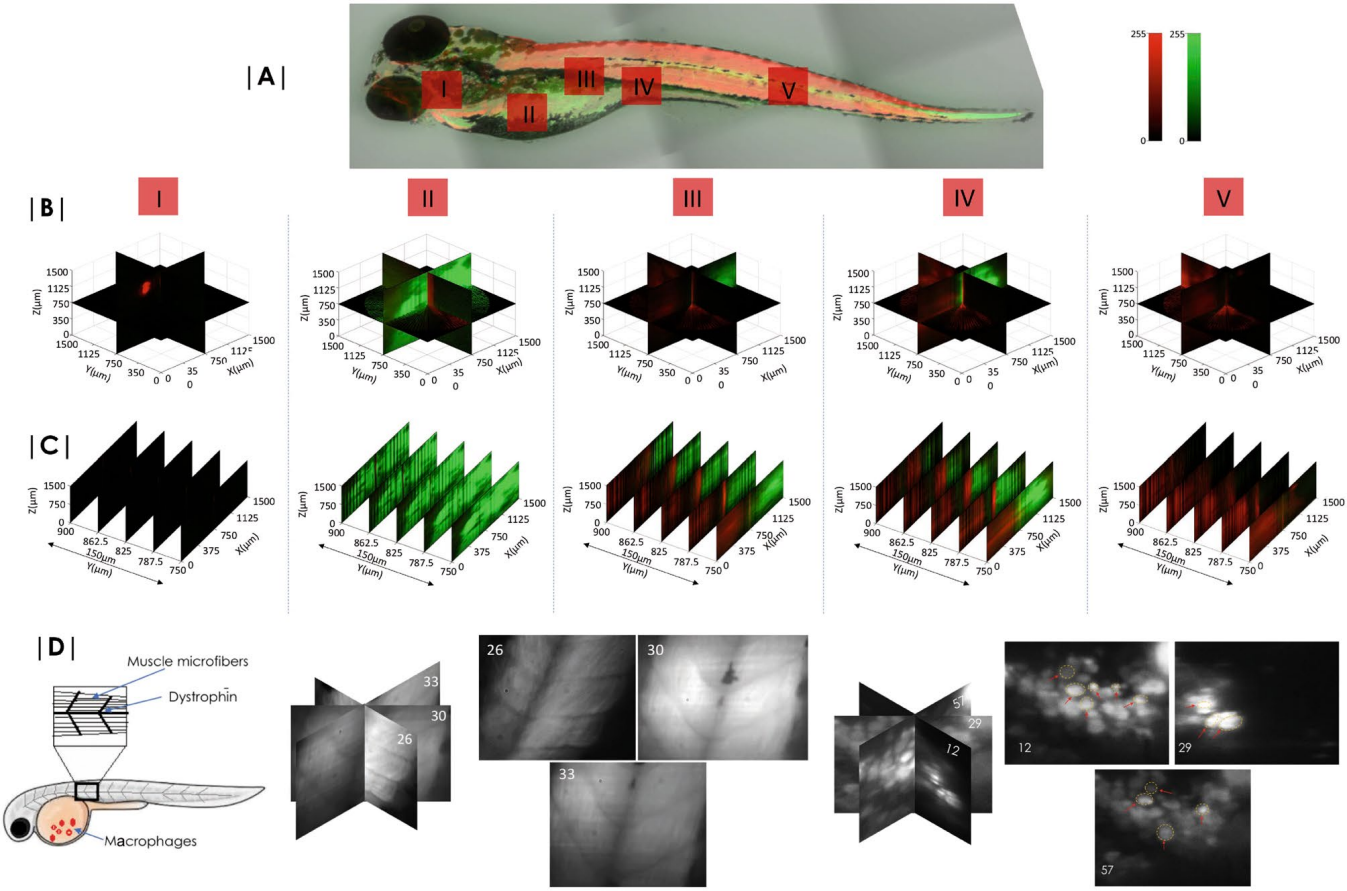
Zebrafish embryo imaging. (**A**) Leica confocal images (tiled) of entire Zebrafish with a 10X objective lens. 48 h Zebrafish embryo with muscles and yolk stained with TRITC and BODIPY respectively show the distribution of lipids and muscles. (**B**) Reconstructed volume images of specific sections (marked I-V) of embryo are also shown. (**C**) 2D slice images from the reconstructed volume. (**D**) Images of muscle structure (planes, 26, 30 and 33) and primitive macrophages (planes, 12, 29 and 57) 24 hours post fertilization. (See, Supplementary 7 for details.

Figure 5 shows the volume image of Zebrafish embedded in agarose-gel matrix. Different sections (marked by I–V in Fig. 5) of Zebrafish were scanned and angular 2D data were recorded. Following reconstruction algorithm (see, Supplementary 3), the volumes of these chosen regions were reconstructed. Simultaneous imaging of two different tissues (muscle and yolk-sac labeled using Phalloidin-TRITC and BODIPY respectively) are evident. Figure 5B shows the volume reconstructed blocks of the section I–V. Few 2D images of the reconstructed volume-stacks are also shown in Fig. 5C to access reconstruction quality visually. Reconstruction quality similar to standard LSM is evident from the reconstructed slices (Fig. 5C). High resolution images of macrophases cluster located in the yolk-sac and muscle Dystrophin with microfiber are shown in Fig. 5D. Details can be found in Supplementary 6.

**Figure 6:**
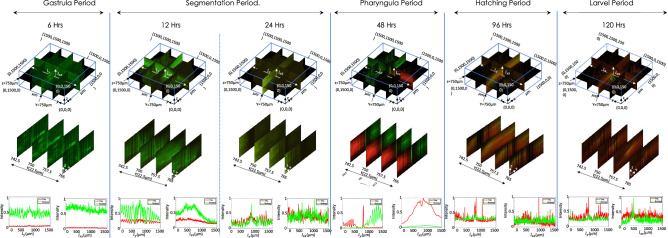
Zebrafish development at various phases indicating a cross-over from lipid-to-protein dependent metabolism. Intensity plot at 6 h period shows BODIPY dominating over TRITC indicating that muscle is yet not developed. At 12 h, there is a rise in TRITC level but dominated by BODIPY, whereas at 24 h significant amount of BODIPY and increased level of TRITC indicates growth of muscles. 48 h volume data shows significant amount of TRITC indicating developed muscles and localised yolk-sac (labeled by BODIPY). Finally, at 96 h and 120 h, BODIPY is localised and distributed over the prominent muscles.

**Figure 7:**
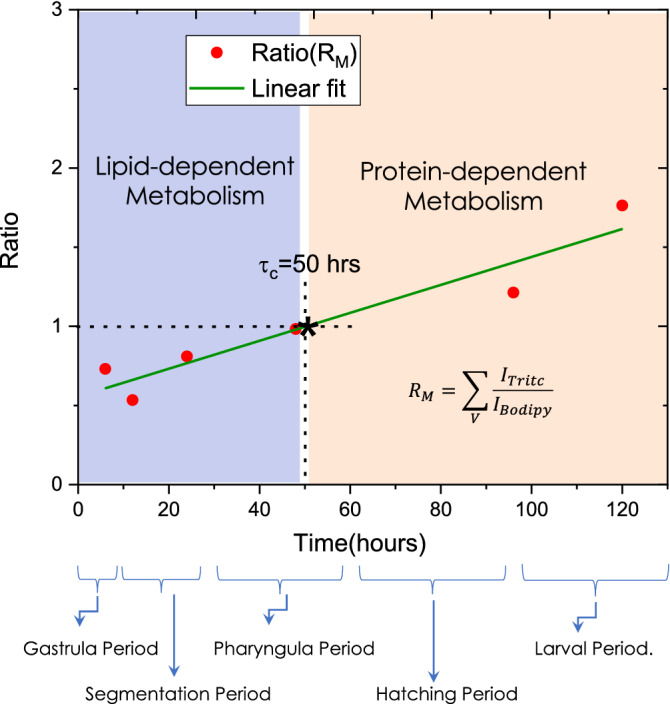
Mean ratio plot for determining the transition from lipid based metabolism to protein based metabolism. The data points are obtained by integrating the ratio of TRITC and BODIPY (representing, muscle and yolk-sac respectively in Zebrafish). A linear fit is used to represent the data. The region to the left of the critical transition time ($\tau _c$) is labeled as lipid-based metabolism and the region to the right of $\tau _c$ as protein-based metabolism. The critical transition time occurs in the Pharyngula period (approximately at 50 h after fertilization).

### Interdependence of muscle development and fat synthesis in Zebrafish embryo

Understanding development of multiple organs at different phases of Zebrafish is critical in Developmental Biology. Muscle development in Zebrafish is particularly interesting as the embryos begin to move very soon after gastrulation. Specifically, muscle development is known to occur somewhere at the interface of Segmentation and Pharyngula period. Moreover, it is reported by Gao et al., that Myostatin is directly responsible for muscle formation as demonstrated by their studies in Myostatin-deficient Zebrafish, thereby indicating a transition to protein-dependent from lipid-dependent energy metabolism^49^. So, the growth-trait of muscle and lipid are strongly inter-linked. This is still ambiguous and calls for further investigation. Using OVSS, we wish to simultaneously visualize and observe the development of muscle and lipid growth in the early stages of Zebrafish.

Rapid volume imaging of Zebrafish at different phases (from day 0– 5) was performed and simultaneous visualization of Muscle and yolk-sac were carried out. Note that, yolk-sac mainly provides the food for the early development of embryos. It acts as the storage of protein and fat apart from Cyanocobalamin (present in yolk-sac), which is essential for fat metabolism and muscle development. Yolk-sac provides the major metabolic fuels for initial embryonic development^50,51^. Figure 6 shows OVSS reconstructed volume stacks of muscle and yolk-sac beginning from Gastrula to the larval period. 3D volume-stacks were reconstructed from the 60 angular data recorded at an angular rotation of $3^\circ$ (following Nyquist Criterion). Datasets were obtained at various time points in development ranging from 6 to 120 h. Reconstructed volume stacks for a small section of the entire Zebrafish along with few selected planes are only shown. It is apparent that, within the first 6 h of the developmental processes, there is no trace of muscle whereas lipid synthesis is active. This start to change at the end of segmentation period where muscle development is initiated in the organism (see, the non-zero intensity of red line plots) while the lipid development process remains dominant. At the beginning of Pharyngula period, the muscle formation is at its peak with lipid development subsiding, since the organism is metamorphosing into its adult stage, which involves active locomotion and feeding abilities. The muscle is completely developed by the hatching period (96 h). Thereafter no further increase is recorded and subsequently the lipid accumulation is in synchrony with muscle development (see intensity-plots for 96 h). In the larval period both the process are fully active.

To understand the development of organs and determine the characteristics energy metabolism at different developmental stages of Zebrafish, we carried out detailed studies in each period. Figure 7 shows the rate of tissue (muscle and lipid) development across all periods. Interdependence Ratio ($R_M = \sum _V I_{Tritc}/I_{BODIPY}$) of volume section containing both muscle tissue (labeled with TRITC dye) and yolk-sac (labeled with BODIPY) is plotted in Fig. 7. This plot interestingly shows the active cycle of lipid- and protein-dependent energy metabolism and its interdependence beginning from Gastrula to Larval period. One can immediately notice the critical time point ($\tau _c$) that represents transition from lipid to protein based metabolism during pharyngula period. The transition (occuring at the interface of blue-orange region) is indicated by the condition, $R \le 1$ (lipid-based metabolism) and $R > 1$ (protein-based metabolism). $R_M$ ratio indicates that lipid and muscle development are strongly correlated and the critical point falls in Pharyngula period at $\tau _c = 50$ h. $R_M > 1$ indicates the dominance of protein-metabolism over lipid metabolism. The slope however is the measure of the relative rate of muscle tissue development. We have also observed structuring of muscle that occur during Pharyngula period and the process steadies in hatching period. However, there is minimal development of new muscle in the Hatching and Larval period. Overall, it appears that $\tau _c$ in pharyngula period is critical in determining the future development of organs. To conclude, OVSS study reveals a transition from lipid-dependent to protein-dependent metabolism in the early stage of Zebrafish development and this occurs at a critical time-point.

## Discussion

The LSM microscopy uses both translation and rotation in tandem to record data. This requires prolonged scanning time for the entire specimen that generates data in the range of Gigabytes to construct a single 3D volume. Long scanning times are prone to sample movement, aberrations and background-fluctuations, and hence are expected to cause severe photobleaching. This rules-out long-time monitoring of multiple organs in real-time. The situation is even more challenging when multicolor imaging becomes imperative. One way to circumvent this is to reduce the data-acquisition time. This can be accomplished by adopting a simplified scanning geometry, enabling parallel multicolor data acquisition and employing dedicated computational techniques to reconstruct multicolor volume-stack in real-time.

In contrast, OVSS technique uses quantized rotation to collect angular 2D image data to reconstruct volume-stack. This eliminates the need for translation and consequently the data recording time is reduced substantially. Since the data obtained is sparse as compared to LSM (that employs both translation and rotation), the reconstruction quality may suffer (see, Supplementary 7). OVSS imaging requires much less data as compared to SPIM and consequently it is much faster. This technique has less number of data points as compared to LSM to reconstruct the entire volume block and so the artifacts are slightly more in OVSS reconstructed volume-stacks. However, this can be minimized by using sophisticated reconstruction techniques (Supplementary 3). In general, volume reconstruction algorithms include coordinate transformation (from cylindrical to Cartesian coordinate system) and quality interpolation methods ($\beta$-spline and higher-order polynomials) for computing data on a regular Cartesian grid. Typically LSFM requires a total multiple-views super-set of $\approx 4000$ images that consumes about 1.1 GB^40^. In contrast, OVSS requires an order-less data i.e., about 180 images and reatively less memory (few tens of MBs). So the availability of fast computing engines and an order-less sectional 2D data may enable volume reconstruction in real-time^52,53^.

Our findings further suggest that phototoxicity is significantly reduced in OVSS microscopy. This is primarily due to reduced light exposure during data acquisition. With an exposure of 10 *ms* per specimen plane, the total time taken for acquiring the entire volume is approximately 2.7 s. On the other hand, LSFM and LITE microscopy use an exposure of 100 ms per image^54^. This translates into $0.1\times 4000$ s (for acquiring 4000 2D angular images) to reconstruct the entire volume stack. Practically, long exposure time bleaches almost all the fluorophores and substantially reduce signal-to-background ratio (SBR). Hence OVSS makes an impact by reducing the total light exposure time thereby decreasing photobleaching and consequently improving SBR of recorded images. However, it may be noted that OVSS is a rapid volume screening technique and may not be comparable to some of the advanced LSFM techniques.

For OVSS imaging, the time to acquire a single angular 2D image of Zebrafish and Drosophila larvae is about 42 ms (10 ms for specimen exposure + 10 ms data-transfer + 22 ms for step-rotation). This makes the OVSS system faster than the existing LSM system clocking with a volume acquisition temporal rate of 0.4 Hz. By increasing the step-rotation speed one can expedite the process substantially. In comparison, IsoSPIM and diSPIM have reported comparable temporal resolution but they are complex, bulky and often require expertise to use the system to its full potential^19,30,39,55^.

The addition of GPUs and GPU clusters along with the proposed quantized rotation-based OVSS system may make the system even faster. It is reported that, GPUs can further accelerate volume imaging^56^. Moreover, the use of multiple CPUs and optionally GPU has accelerated the reconstruction process in less than a 15 min on a NVdia P6000 graphics board^57^. Specifically, Biobeam computational software based on GPU implementations has substantially reduced volume reconstruction time^56^. Current state-of-the-art light-sheet microscopes requires a lot of precious data-acquisition time that severely slows down the imaging process^58,59^. Another issue is related to chromatic abberation introduced by light of different wavelengths used for excitation. OVSS overcomes these limitation by employing overlapping dual-color light-sheet for illumination and using discrete-rotation based isotropic data acquisition. We anticipate that OVSS system integrated with GPUs can reconstruct volume image in a record time never realized before.

We have designed and demonstrated a simple yet effective microscopy system for studying the growth of rapidly developing tissues in Zebrafish and *Drosophila*. Both single and multi- color OVSS imaging were carried-out to determine the functioning and development of organs during different phases. Imaging of Drosophila larvae is demonstrated using multicolor OVSS microscopy (see, Supplementary 8). The neurons and body fat of an intact *Drosophila* third instar larva are respectively labelled with DsRed and Bodipy respectively. Three different regions of *Drosophila* are shown in Supplementary Fig. S8-1 (Supplementary 8). The quality can be accessed from the reconstructed volume, given the fact that chitinous covering is not removed. Subsequently, multicolor OVSS imaging is demonstrated on Zebrafish embryo samples, and image-based quality assessment is carried out (see, Fig. 4). A more advanced design was adopted for observing multi-organ growth at different phases of Zebrafish (Fig. 6). This design required integration of an additional laser ($\lambda = 532$ nm) to excite fluorophores in a different spectral window. The fluorophores (TRITC and BODIPY) were chosen to ensure that their emission spectra do not overlap and two distinct spectral emission windows (green window (488–512 nm) for 475 nm excitation and red window (550–600 nm) for 532 nm excitation) were considered. This minimizes bleed-through in the red channel and thus ensures spectral separability of the fluorescent dyes. It may be noted that we have avoided water-dipping objectives, instead we employed air-objectives. This simplifies the sample mounting chamber which is critical to light-sheet microscopy. Of course air-objectives give rise to optical aberrations. To circumvent this effect, we have used an iris in the detection sub-system to cut-off the light emanating from non-focal planes. This has substantially reduced optical aberrations. The details are discussed in Supplementary 5. In addition, we added two Supplementary Videos 1 and 2 of gel-embedded fluorescent beads without and with iris in the detection path, respectively.

In general, optical imaging including light-sheet microscopy faces an unavoidable trade-off between resolution and depth of field (DOF). High resolution requires objectives with high numerical apertures (NA), but the associated large angular uncertainty results in a limited range of depths that can be put in sharp focus.

The multicolor advantage coupled with parallel data acquisition is probably the best choice for simultaneous study of multiple organs during development. With the aid of multicolor OVSS imaging, we followed the growth of muscle and yolk-sac in Zebrafish beginning from embryo to adult stage. We found an interesting correlation and interdependence between the growth pattern of filamentous actin (reflecting in muscle growth) and lipid depletion (in the yolk-sac) as the Zebrafish transition from one to another period (See, Figs. 6 and 7). Study reveals transition from lipid-dependent metabolism to protein-dependent metabolism in the early stages of development (Pharyngula period).

Our method enjoys the dual advantage of being amenable to faster data generation, and preserving the context of inter-tissue and inter-organ relationships, that are lost during larval dissections. This will be a vital technique to decipher how different proteins distribute during developmental processes, and how one organ system depends on another. In future, OVSS imaging may facilitate comparison of gene expression patterns, and tissue connections, between wildtype and mutant organisms.

OVSS imaging integrates the benefits of fast multicolor imaging (provided fast computing engines are employed) with minimal photobleaching, an order-less memory requirement and simultaneous multi-organ volume interrogation. Such a system is helpful for long-time monitoring of rapidly occurring biological processes and interdependent organ study during early development.

## Methods

### Optical setup

OVSS system has three major sub-systems: multicolor lightsheet illumination, automated sample chamber and simultaneous dual-color detection :

*Multicolor lightsheet illumination* Simultaneous illumination of multiple organs (labeled with spectrally distinct fluorophores) require separate light sources. Depending on the fluorophores (here, TRITC and BODIPY) used in the present study, two different lasers (Laserglow, 532 nm, LCS-0532-TDD-00100-05, Avg power=207.2 mW) and 473 nm Laserglow, LRS-0473-TSM-00100-10, Avg power=124.1 mW) were used. The beams were directed by mirrors and combined using beam-combiner dichroic mirror (DM). Linear graded neutral density filters (Thorlabs, USA) were used to match the beam intensity at the specimen. The laser intensity used at the specimen was about 3.4 mW. The green light was made to pass through an unit-magnifier (that is placed on a linear-translator) in order to co-align the diffraction-limited lightsheets. The combined beam was expanded and passed through a cylindrical lens to form a lightsheet. A 10$\times$ objective lens (Olympus 0.3 NA), is placed at the focus of cylindrical lens to form a coplanar two-color diffraction-limited light sheet.

*Motorized sample chamber* The sample was embedded in a Agarose gel-matrix and placed inside a capillary tube. The tube is placed on a pre-fabricated hole on the rotational stage (Holmarc Inc., MRS-50) which is motor-controlled (see Supplementary 1 for control system design). The control is achieved using Arduino board (Arduino Uno) and Arduino program is developed to control rotation with a resolution of $3^\circ$. It may be noted that, proposed OVSS imaging requires specimen centering to avoid refocussing and necessary correction for positional shifts. Additional translation stages (Holmarc Inc., MTS-65-65-1) are integrated with the rotation stage in order to place the sample at the focus of multicolored light-sheets. The photograph of the actual experimental sample chamber is shown in Fig. 1 and Supplementary 1.

*Dual-color detection* For detection orthogonal detection system is developed. The emitted light from the specimen is collected by the detection objective (Olympus 4X, 0.1 NA) and made to pass through an Iris to cut off the spherical-aberrated rays. Notch filters [Green notch filter (ZET532NF, Chroma) and Blue notch filter (ZET473NF, Chroma)] were used to remove scattered and reflected excitation light. Subsequently, a combination of tube lens (focal length, $f=200$ mm, Thorlabs) and dichroic beam-splitter (cut-off=505 nm, Thorlabs) was used to focus and separate green/red fluorescence. In front of green signal detector, band bass filter(Edmund optics, Bandpass $500\pm 12$ nm) is placed to collect only required signal. Similarly in front of CCD2 detector long pass filter of cut off 550 nm (Thorlabs) is used to collect red fluorescence. The CCD Chameleon cameras (CMLN-13S2M-CS, pixel size $3.75~\upmu$m) were purchased from Pointgray.

### Biological sample preparation protocols

#### Multicolor bead sample preparation

To prepare the Agarose gel-matrix, 1 g of agarose was dissolved in 100 ml of distilled water to prepare $1\%$ gel. The mixture was boiled at $121 \;^\circ$C and subsequently filtered using syringe filter (Biofil) while it is still hot. The solution is then allowed to cool at $50\;^\circ$C. $20~\upmu$L of both the beads are mixed and resuspended in 1 mL of liquified agarose solution. The beads were purchased from Invitrogen with specific excitation-emission characteristics. They are, Invitrogen FluoSpheres Amine-Modified Microspheres, $1.0 ~\upmu$m, yellow-green fluorescent (505/515) ( Catalog number: F8765), and Invitrogen FluoSpheres Carboxylate-Modified Microspheres, $1.0 \; \upmu$m, Nile Red fluorescent (535/575), (Catalog number: F8819). The filtered liquid mixure is then poured in a capillary tube (Z114995, SigmaAldrich) of volume $100--200 \upmu$L) and left to dry for 10 min at room temperature.

#### Preparation of single and multicolor Zebrafish embryo

The Zebrafish (Danio rerio) was used for multicolor imaging. Zebrafish is remarkably similar to the human in its body plan, organ composition and physiology, and is easy to manipulate genetically^60^. It is used to study developmental biology and human diseases^61^. To prepare single color Zebrafish embryos, fertilized embryos were fixed for 30 min in $4\%$ paraformaldehyde solution in PBS, followed by extensive wash in PBS. Embryos were then permeabilized with $0.1\%$ TRITON X-100 in PBS (PBTX). Then embryos were stained with $12~\upmu$g/mL TRITC-phalloidin (Sigma-Aldrich) conjugate solution in PBTX for 1 h at room temperature in dark. Subsequently, embryos were washed with PBS thoroughly. To comprehend the relation between reduction of yolk-sac and development of muscle we carried out experiment with fertilized embryos of Zebrafish. Various stages of fertilized embryos were taken starting from 6 to 120 h. The paraformaldehyde fixed samples were used at different developmental stages of choice. For such studies, a number of imaging techniques exist that successfully allow recording of similar data at higher quality. We demonstration our study on fixed Zebrafish embryos after every stage. However, it may be possible to image specimens for longer periods of time (about a day) to access organ differentiation. Please note that, imaging live specimens is technologically challenging due to sample movement. This may further degrade image quality and takes away the advantage of light sheet imaging. We have used phalloidin-TRITC and Bodipy to demonstrate multicolor OVSS volume imaging system. However, long-term phalloidin staining may cause toxic effects (such as, muscle fibre differentiation) and such studies can only be done in living developing organisms.

For multicolour staining of Zebrafish, two distinct region were selected which is biologically known to be active during early developmental. We chose BODIPY 493/503 (Invitrogen-D3922) a green fluorescent lipophilic dye which stains for neutral lipids and can be used as a tracer for oil and other nonpolar lipids. Yolk is the major region of Zebrafish which is stained by this dye and gives distinguishable pattern. BODIPY (D3922, Molecular Probes, Carlsbad, Calif, USA) (excitation wavelength 480 nm, emission maxima 515 nm, was diluted in PBS or DMSO and stock concentration of 2 mg/mL prepared. The stock DMSO solution is diluted 1 : 300 into DMEM (Gibco, 21063029-No phenol red). The other part is muscles which are stained with TRITC-phalloidin that binds actin of muscles in Zebrafish embryos.

For multiple staining, first embryos were fixed in $3.7\%$ formaldehyde followed by PBS wash. Then, TRITC-Phalloidin staining was done. Embryos were incubated at room temperature for 1 h. It is then followed by counterstaining with BODIPY 493/503^62^. Embryos were stained with 1:300 BODIPY and incubated for 1–1/2 h. After this, the embryos were washed with PBS. Zebrafish embryos were transferred into the imaging chamber/capillary tube (Z114995, SigmaAldrich) of volume $100-200 ~\upmu$L. The capillary tube was first filled with filtered liquefied $1\%$ agarose gel. Then with the help of pipette Zebrafish was gently placed inside the capillary tube. The end of capillary tubes were subsequently sealed using adhesive.

### Imaging system automation and data acquisition

In OVSS imaging system, raw image data are recorded using a dual CCD camera based detection system (see, Fig. 1). The data obtained in each channel (Green and Red channels) were subjected to volume reconstruction. The processing steps include, registration, interpolation, transformation and fusion. To ensure synchronized data collection, stepper motor automation and triggering CCDs is achieved via Arduino environment. Specifically, Arduino Uno control board is used and all the devices (stepper motors, CCDs) were triggered using a single clock-pulse of duration 10 ns via respective trigger connection pin. An Arduino code was written to control and synchronise between stepper motor degree of rotation and triggering CCDs. The camera was triggered in the mode 0 by using the GPIO pins as external trigger. When the camera is put into Trigger Mode 0, the camera starts integration of the incoming light from external trigger input falling/rising edge. The Shutter/exposure value describes integration time. In synchronisation timing we ensured two things firstly as soon as rotations is done the shutter integration starts and secondly next shutter integration should start only after complete data transfer. More details about the automation is in electronics connection and synchronisation timing diagram is given in Supplementary 1.

For accurate registration of multi-channel data correlation function along with fluorescence bleedthrough mechanism was exploited. Fluorescent microbeads (*Ex*/*Em*:  505 nm/515 nm) of size $1~\upmu$m were used and a bleedthrough of fluorescence (in the Red channel) was observed at high power ($\approx ~200$ mW). The raw data (2D angular image) thus recorded were subjected to cross-correlation analysis in MATLAB and the shift-map in image registration was computed. A sample correlation-map is shown in Supplementary Fig. S2-1 (Supplementary 2). This map is used to co-register the data obtained from both the channels. We have used MATLAB inbuilt functions for coordinate transformation and interpolation. Registration, transformation and fusion were implemented in MATLAB2018b on operating system 64-bit windows 10 of hardware i-Mac, dual Intel-core-i5-2.5 GHz, 4 GB RAM, 500 GB storage. Before registration, the sectional angular images were filtered with gaussian 3 $\times$ 3 kernel filter of variance 0.9 to reduce noise. The raw YZ image data were combined in a computer cylindrical coordinate system (this mimics sample in a capillary tube). This is followed by transformation to regular cartesian system and MATLAB inbuilt spline-interpolation function was used. Finally, the volume data thus obtained in cartesian system were merged to obtaion the multicolor volume-stack. Detailed discussion on data acquisition and volume reconstruction protocols can be found in Supplementary 3.

### Ethical approval

All the experimental procedures were reviewed and approved by the Institutional Committee (IC), Indian Institute of Science. We declare that, all methods were carried out in accordance with relevant guidelines and regulations approved by Institutional Committee (IC), Indian Institute of Science.

## Supplementary Information

Supplementary Video 1.

Supplementary Video 2.

Supplementary Video 3.

Supplementary Video 4.

Supplementary Information.

## Data Availability

The codes, datasets, Videos and 3D reconstructed volume stacks acquired for this study are available from the corresponding author upon request.
