# Supplementary material for: Fluorescence based rapid optical volume screening system (OVSS) for interrogating multicellular organisms
Source: Sci Rep. 2021 Apr 7;11:7616. doi: 10.1038/s41598-021-86951-3 (PMC8027194; doi:10.1038/s41598-021-86951-3)
Supplement: Supplementary file 5 — Supplementary Information. [file 41598_2021_86951_MOESM5_ESM.pdf]

Title: Fluorescence based Rapid Optical Volume Screening System (OVSS) for Interrogating Multicellular Organisms

Authors: Jigmi Basumatary, Tarannum Ara, Amartya Mukherjee, Debanjan Dutta, Upendra Nongthomba and Partha Pratim Mondal

Supplementary 1 – 9

Supplementary 1: System Automation and Synchronisation

Supplementary 2: Image correlation

Supplementary 3: Data Collection and Volume Reconstruction Protocol

Supplementary 4: Photobleaching Study

Supplementary 5: Tackling Optical Aberration in OVSS Microscopy

Supplementary 6: High Resolution Raw Images and Volume-Stack Using 10X/0.3NA Detection Objective in OVSS System

Supplementary 7: Shannon's Entropy : Measure of Information Content

Supplementary 8: Drosophila Larvae Imaging

Supplementary 9: Time-Lapse Imaging

Supplementary 1: System Automation and Synchronisation

Rapid volume imaging requires automation of entire imaging system for synchronized data collection. For OVSS system, Arduino Uno controller is used [1]. Easy programming-based control, low cost and less prerequisite makes it a system of choice for control and automation. Ranging from controlling the linear and rotational motors to synchronized camera detection in multiple channels is achieved by Arduino Uno board.

Arduino Uno [1] which uses ATMega328, an 8-bit microcontroller with most instructions running at one instruction per clock using a 16MHz crystal. The camera used 12-bit A/D converter and has speed of 18fps at 1296x964 pixels of size 1201kb, hence data transfer rate of ~21.6mb/sec, an exposure range 0.01ms to 10 seconds. CCD has USB 2.0 interface.

Sample holder shaft is attached to the stepper motor with gear ration of 1:72. Stepper motor speed is set to 26rpm with 4000steps/rev with a resolution of $0.9^0/step$. Therefore for $\sim 3^0$ (i.e., 800steps) displacement of shaft it takes $\sim 21ms$. This implies that the data can be acquired at a rate of 7Mb/ms whereas in our actual case we acquired data transfer of ~218.4kb/ms. This means ~1201kb can be transferred at a time of ~5.5ms. Hence in terms of cycle acquisition rate is 1201kb/cycle.

To ensure synchronized data collection at both the CCDs, a single trigger is used on both CCD.

Control Circuit Diagram

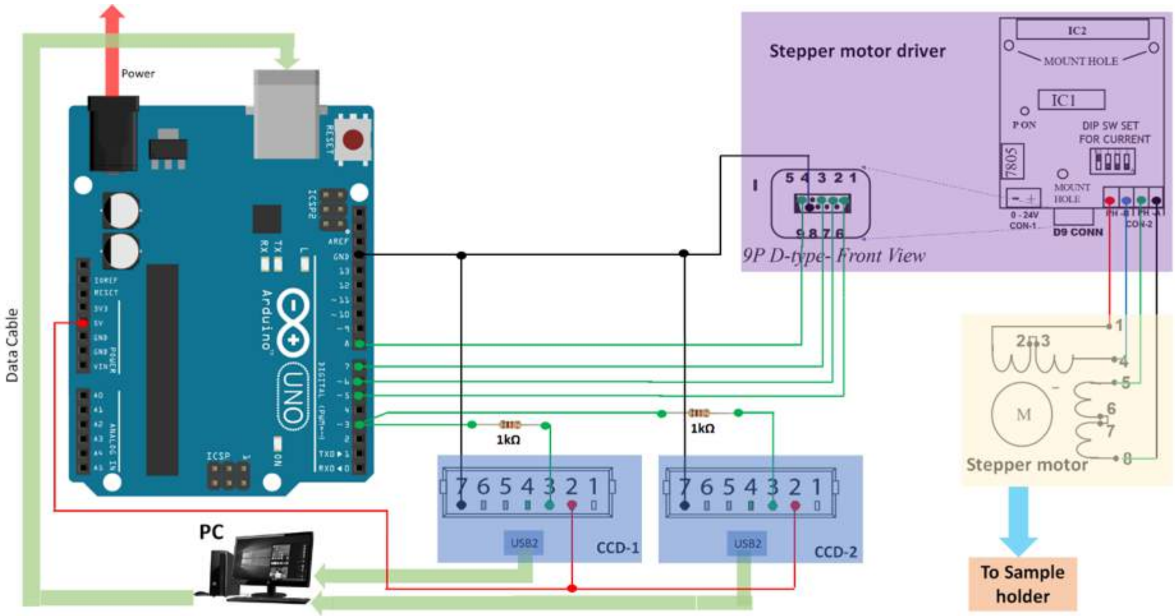

Fig.S1. Automation Schematic circuit for controlling and synchronizing our imaging system. Arduino drives the Stepper motor is driven via stepper motor driver (NSK electronics). To avoid damage of camera a resistor of$1k\Omega$ is used. Both the cameras are connected to Pin 3 for synchronization.

Actual Setup Diagram:

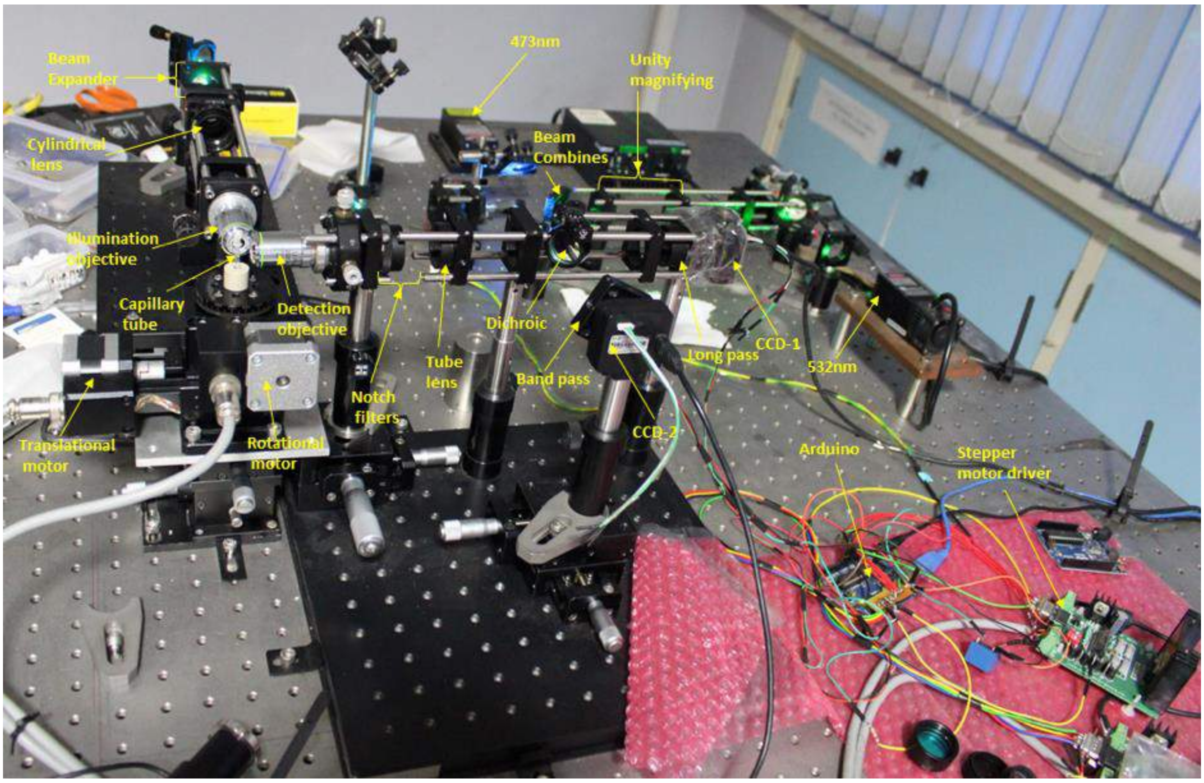

Timing Diagram:

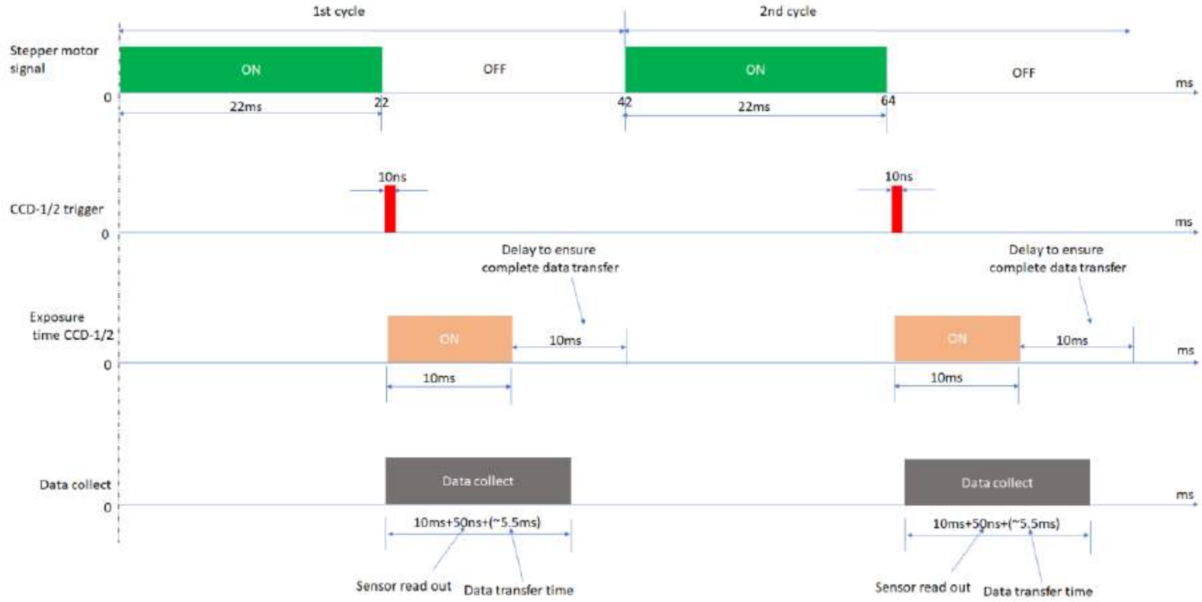

Fig. S2. Automation synchronisation timing diagram for CCD-1 and CCD-2 and data acquisition for 3^0 . Initially rotational motor is rotated 3^0 followed by triggering signal of pulse width 10ns. Exposure/shutter value is 10ms. This gives data collect time of approximately 16ms. To ensure complete data transfer to PC, exposure time of 10ms and data acquisition of 10msec to include additional delay will result in a total time

of 22msec is introduce before next cycle stars. In one cycle we are acquiring one frame of image 1201kb/cycle.

The CODE

%%%Aurdino code for Synchronous Data Collection by CCD-1 and CCD-2 %%%

```
#include <Stepper.h>                          //calls inbuilt function for stepper
int for_rot = 4000;                           // defines rotational steps in one revolution
int speed_rot_for=26;                         //defines speed of stepper motor in rpm

Stepper motor_for_rot(for_rot,5,6,7,8);       // define pin no for motor connection
int n=3 ;                                     // select degree you want to rotate
int s;
int z=360/n;                                  //no of images to be captured in one
revolution

void setup() {
motor_for_rot.setSpeed(speed_rot_for);        //set stepper motor speed
digitalWrite(5, LOW);                         //set pin no at low
digitalWrite(6, LOW);
digitalWrite(7, LOW);
digitalWrite(8, LOW);
pinMode(3,OUTPUT);                            // camera trigger pin
}
void loop() {
for(s=0;s<(z-1);s++)
{
delay(10);
motor_for_rot.step(800*n);                    //rotate motor
digitalWrite(3,HIGH);                         //camera trigger ON
delay(10);                                    //Camera exposure time
digitalWrite(3,LOW);                          //camera trigger OFF
}
exit(0);                                      // exit from lopping
}
```

References

[1] https://robu.in/product/updated-version-beginners-starter kit/?gclid=CjwKCAjw7anqBRALEiwAgvGgm06NZi4FxweRMJI IPbda6EkWj8HPK_FCzQdj25rU9fB3NIzDLjh7xoCo5AQAvD_BwE

Supplementary 2: Image correlation

Multicolor OVSS imaging heavily depends on accurate overlapping of image data obtained from two synchronized channels (Green and Red channel). This requires correlation analysis of both the channels with a known sample. We have used Green fluorescent beads suspended in Agarose gel-matrix (see, method section-B.1) and the bleed-through mechanism (at large exposure times) to correlate data obtained from both the channels.

Normalised correlation technique between reflected green fluorescence signal and its bleed through is used to ensure the processing of the same section of two-colour images. Green fluorophore reflected green signal and its bleed-through images of 1280x960 pixels (of size, 3.75μm) is recorded. Bleed through signal is taken as reference for processing. The following short protocol is followed:

(1) Reflected green fluorescence signal is to be rotated by 180°. This ensures mirror image of the reference signal.
(2) The mirror image is vertically flipped for same orientation as the reference.
(3) Rotation axis is identified (see, Supplementary 8) and about rotation axis useful image area called as templet is selected.

$$\gamma(u,v) = \frac{\sum_{xy}[\, f(x,y) - \bar{f}_{u,v}\,][\, t(x-u,y-u) - \bar{t}\,]}{\sqrt{\sum_{xy}\left[f(x,y) - \bar{f}_{u,v}\right]^2 \sum_{xy} t(x-u,y-v) - \bar{t})^2}} \text{.............}(1)$$

where, $\bar{t}$=mean of template.
f= target image.
$\bar{f}_{u,v}$ = the mean of $f(x,y)$ in the region under the template.

Consider f(x,y) and t(x,y)are the two images to be corelated representing the template and target image respectively. The target image i.e., reflected green fluoresce is placed over the template image and normalized correlation coefficient [1] for each pixel in the template image is found using equation (1) to construct the correlation map. After sliding the target through all the pixels in the template image, the maximum coefficient and its pixel position with is obtained. In terms of pixel position of maximum correlation coefficient and size of templet, the matched templet on target can be found. Fig.S3 shows the complete correlation process of yellow green bead of size 1μ, with reflected image on CCD-1and bleed through image on CCD-2 as input data images.

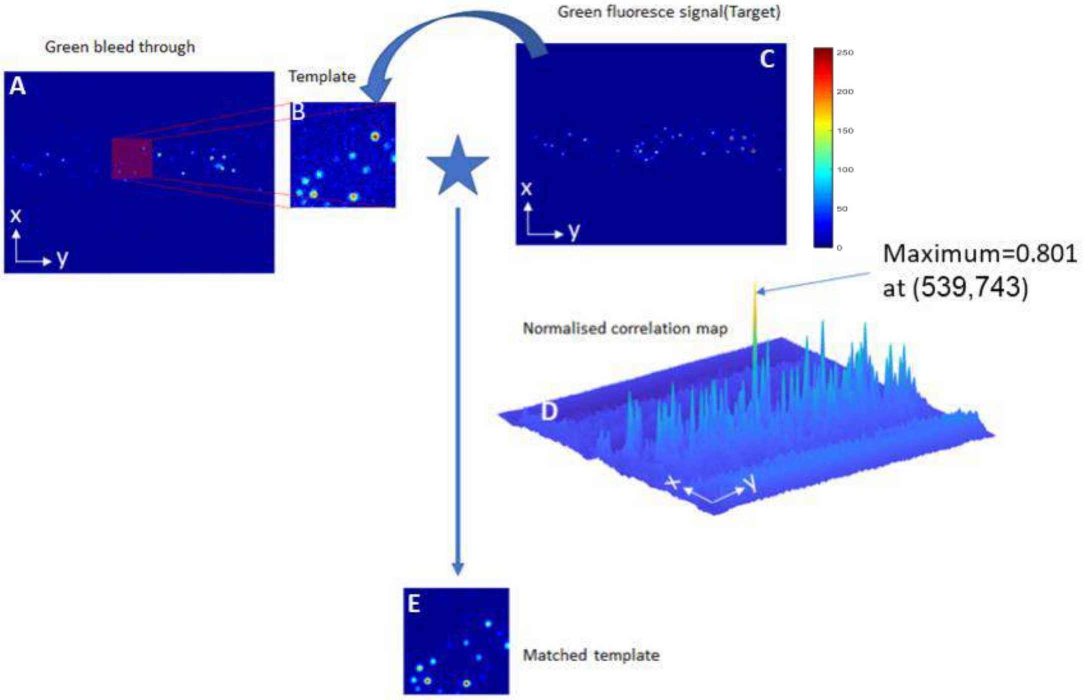

Fig.S3: Image selection correlation to ensure processing of same section data. (A) Green bead bleed through image on CCD-1(B) About rotation axis a section green bleed through image is selected as a template. (C)Green bead image on CCD-2 slides over template and corelates. (D) Normalised correlation map, maximum correlation value is identified and using corresponding index (E) matched template is extracted from (C) taking the maximum correlation location as origin.

Reference

[1] Lewis, J. P., "Fast Normalized Cross-Correlation," Industrial Light & Magic.

Supplementary 3: Data Collection and Volume Reconstruction Protocol

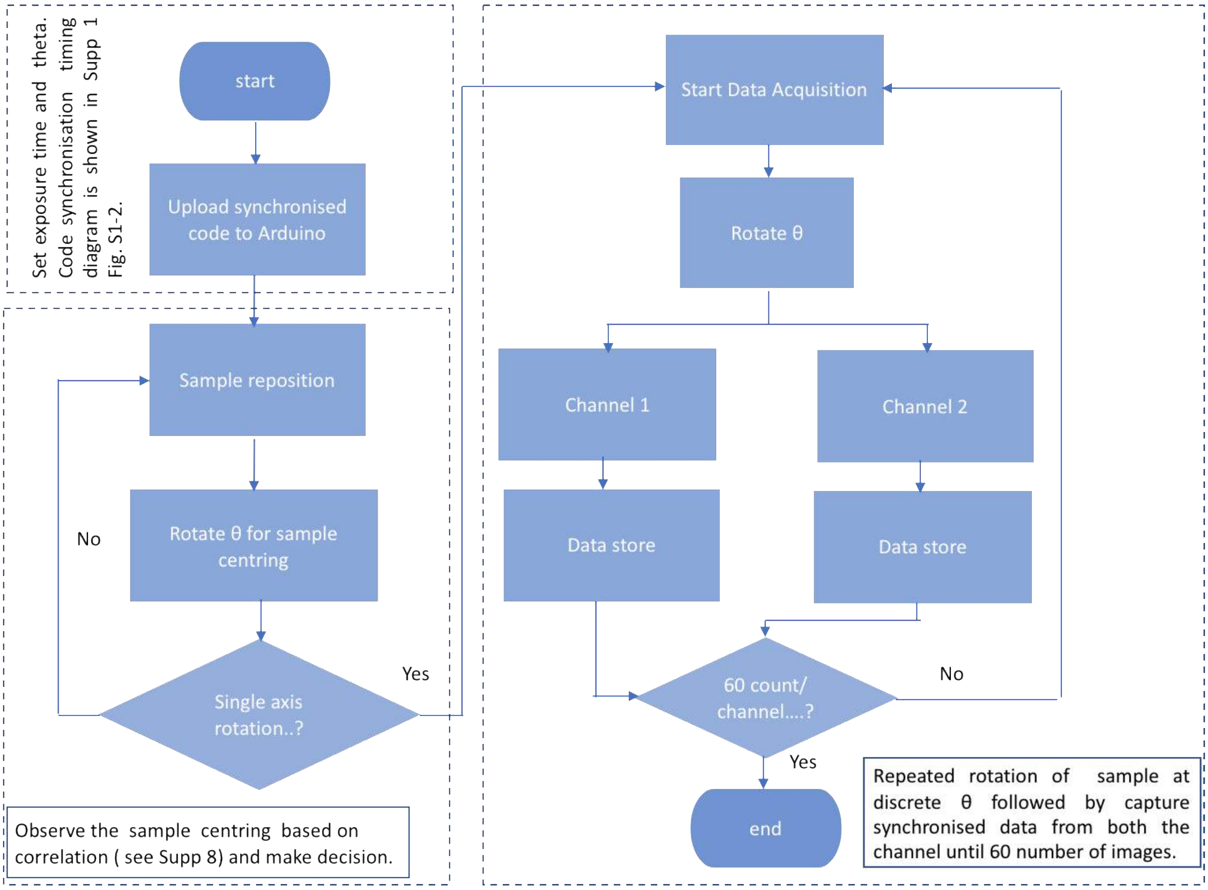

Fig. S4: Data Recording Protocol for synchronized data collection in both the detection channels.

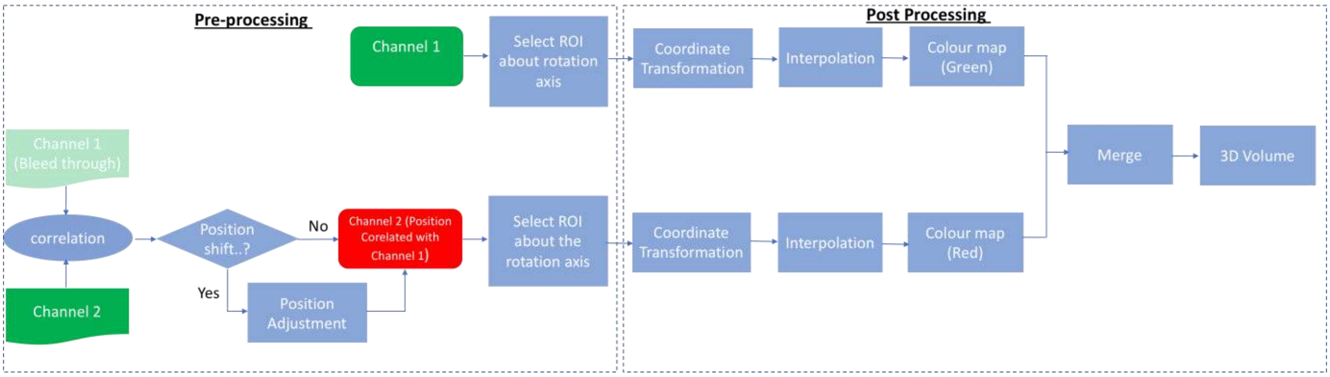

Fig. S5: Volume reconstruction protocol that incorporates correlation, transformation and interpolation.

The raw images are obtained at an angle and so we call it angular images. Since images are recorded in cylindrical coordinate system, we need to transform them to Cartesian Coordinate system. This calls for a couple of mathematical operations: (1) Cylindrical to Cartesian transformation and, (2) Interpolation to determine the values on cartesian grid.

1. The transformation from Cylindrical to Cartesian System for any point (x,y,z) in Cylindrical System (Sample in Cylindrical Capillary tube) is given by,

$$\begin{bmatrix} x' \\ y' \\ z' \end{bmatrix} = \begin{bmatrix} cos\theta & -sin\theta & 0 \\ sin\theta & cos\theta & 0 \\ 0 & 0 & 1 \end{bmatrix} \begin{bmatrix} x \\ y \\ z \end{bmatrix}$$

   where, θ is the angle.

   Figure S6A shows some of the angular raw images (for both Green and Red beads) recorded by CCD camera. Subsequently, Fig.S6B shows the exact position of these images in cylindrical coordinate / Capillary tube sample.

2. The Interpolation from Cartesian (Random) points (x',y',z') to regular Cartesian point (x'', y'', z'') has been achieved by the Cubic interpolation method.

   Cubic interpolation is carried out to determine the pixel value at regular grid points. To determine the value at a regular point (x'',y'',z''), four neighbouring known values (say, P_0, P_1, P_2, P_3) are used. The value at the regular grid point is given by,

$$f(x) = a + bx + cx^2 + dx^3 \dots\dots\dots\dots\dots\dots\dots\dots\dots\dots\dots\dots\dots (1)$$

   where a, b, c and d are constant and P_0, P_1, P_2, P_3 are related as,

$$\begin{cases} a = -\frac{1}{2}P_0 + \frac{2}{3}P_1 - \frac{3}{2}P_2 + \frac{1}{2}P_3 \\ b = P_0 - \frac{5}{2}P_1 + 2P_2 - \frac{1}{2}P_3 \\ c = -\frac{1}{2}P_0 + \frac{1}{2}P_2 \\ d = P_1 \end{cases}$$

The hardware system used for data processing consist of iMac i5 -2400S, 52.50Ghz,52.50Ghz 4GB RAM, at 3 degree sampling total 60 2D angular image with 400 x 400 pixel size. The post processing time for volume reconstruction is about, (3.4(reading data)+308.37(coordinate transformation)+180.69(interpolation))*2(two channel)+50seconds(merging)=16.9minutes.

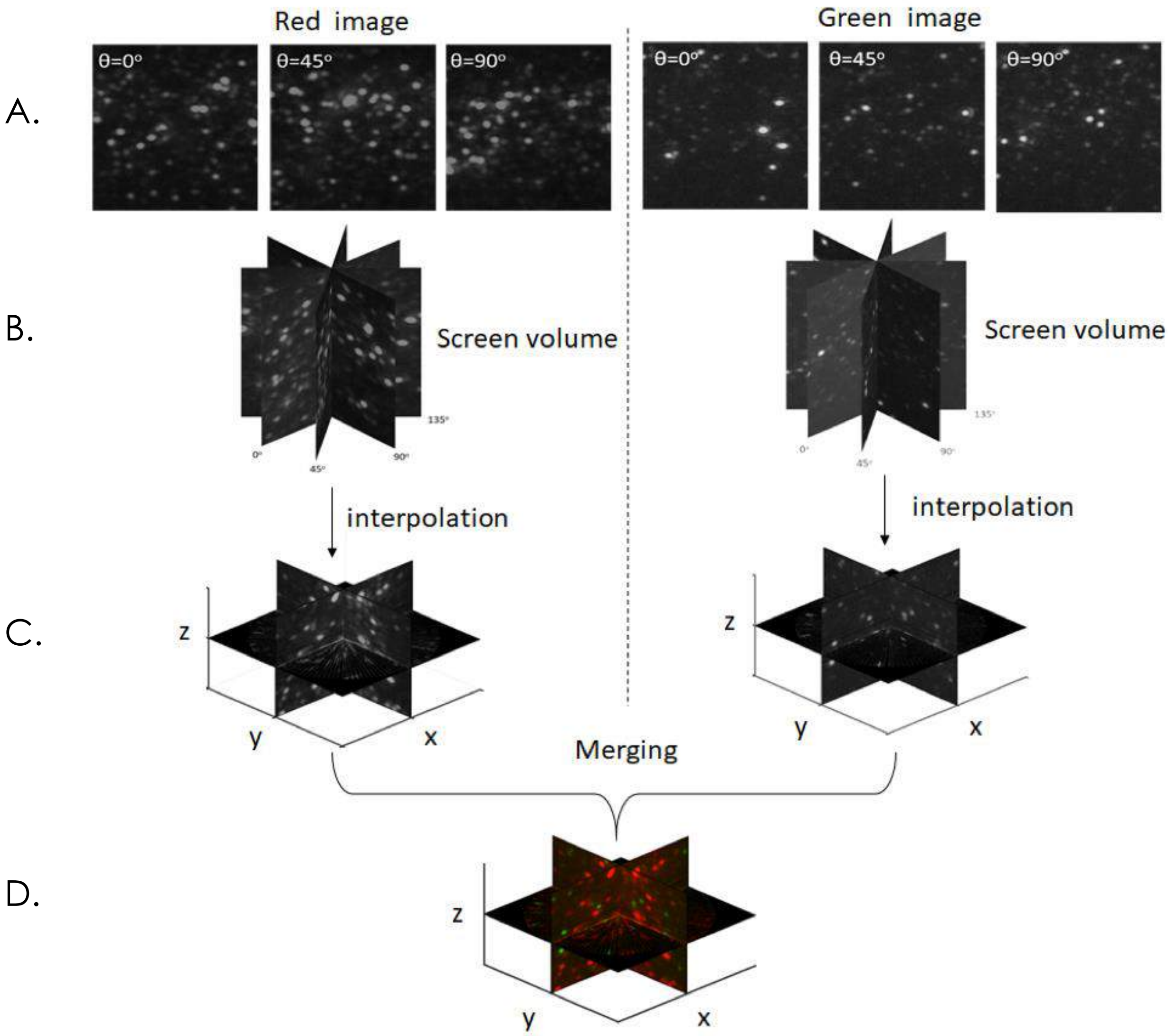

Fig.S6. (A) Raw recorded 2D data at different angles θ. Left: - 1μ size Nile Red bead (Ex=535nm/Em=570nm) on CCD-1. Right: - 1μ size yellow green beads (Ex=505nm/Em=515nm) on CCD-2. (B) Using 2D recorded data constructed 3D screening volume image, left: - Nile Red beads Right: - Yellow green beads (C) Interpolated complete volume images, left: -Nile Red beads Right: - Yellow green beads (D) Merged colour image where Nile Red beads is assigned red colour and yellow green beads is assigned green colour.

We observed that, the data processing that involve rotational data acquisition and subsequent cubic interpolation hampers the quality of volume reconstruction process. This is further evaluated in terms of information content (Entropy measure) as discussed in Supplementary 7.

Supplementary 4: Photobleaching Study

Multicolor volume imaging requires prolonged exposure to record volume stack. But long exposure causes severe photobleaching and ultimately the image quality suffers. The image contrast further deteriorates when imaging for prolonged duration is desired [1][2]. Potential ways to overcome bleaching is to accelerate the data acquisition process and minimizing light exposure. Prolonged exposure to light weakens the ability of fluorophore to fluoresce. This becomes even more challenging in applications that require long-time monitoring [1][2][3]. Often one tends to increase the intensity of incident light to compensate for weak fluorescence signal but that leads to fast photobleaching. In practice, countering bleaching for prolonged imaging requires judicial use of exposure time during data acquisition.

Fig.S7 shows the characteristic photobleaching of fluorophores as a function of time in Zebrafish embryo and *Drosophila* larvae. The yolk-sack of Zebrafish embryo is labelled with BODIPY that binds to lipids and the muscle is labelled with TRITC which is an Actin-binding fluorophore. In *Drosophila* larvae, the nervous system expresses DsRed(T4) and lipid-containing fat body are stained with BODIPY. Standard photobleaching experiment was carried-out to understand the characteristics of these fluorophores in physiological conditions. The raw data thus recorded is fitted to the exponential function (following exponential decay law, $I = I_0\, e^{-t/\tau}$, where, τ is the bleach-time constant) that reproduces the exponential decay typically associated with photobleaching [4][5]. From the fit, we deduced the bleaching time-constant to be τ =110 secs for TRITC (ex/em =557nm / 576nm), τ =5 secs for BODIPY (ex/em =493nm / 503nm) and τ = 60 secs for DsRed(T4) (ex/em =555nm / 586nm). The bleach-time constant indicate that, the fluorescing ability of fluorophores fall to 1/e of its initial value (at t=0). The effect of bleaching in Zebrafish embryo and *Drosophila* larvae for the first 400 secs are also shown in Fig.S7.

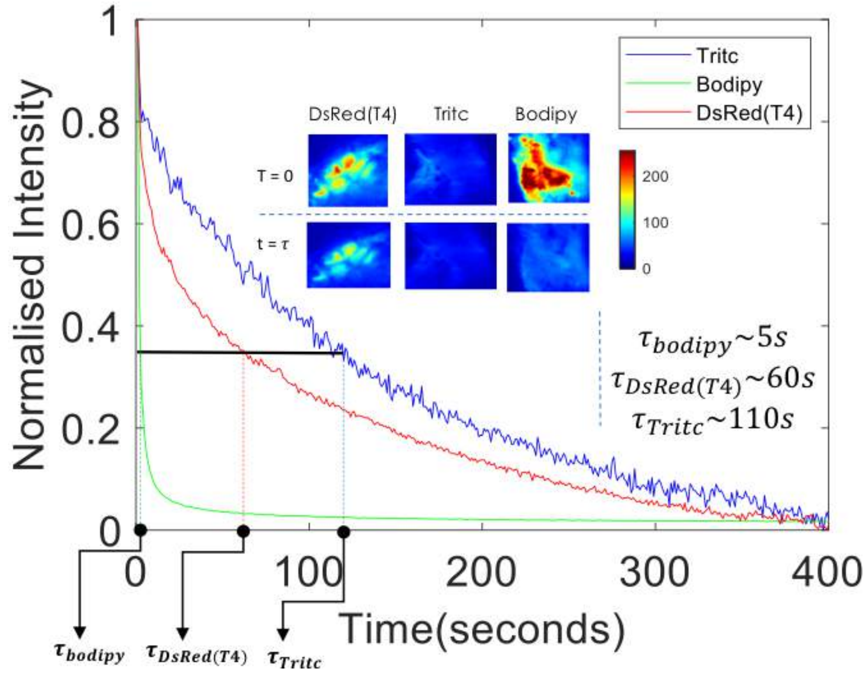

Fig. S7: The study of photobleaching characteristics of BODIPY, DsRed (T4) and Tritc that are used to label and quantify organ development in Zebrafish embryo and *Drosophila* larvae.

References:

[1] T. Bernas, B. P. Rajwa, E. K. Asem, J. P. Robinson, J. of Biomedical Optics, 10(6), 064015 (2005).

[2] Hoebe, R.A., C.H. V. Oven, T.W.J. Gadella Jr, P.B. Dhonukshe, C.J.F. V. Noorden and E.M.M. Manders.: Controlled light-exposure microscopy reduces photobleaching and phototoxicity in fluorescence live-cell imaging. Nature Biotechnology, 25 (2), 249-253 (2007).

[3] B. Munkhbat, M. Wersäll, D. G. Baranov, T. J. Antosiewicz and T. Shegai, Suppression of photo-oxidation of organic chromophores by strong coupling to plasmonic nanoantennas, Science Advances, 4, eaas9552 (2018).

[4] L. Song, E. J. Hennink, I. T. Young, and H. J. Tanke, Photobleaching Kinetics of Fluorescein in Quantitative Fluorescence Microscopy, Biophysical Journal, 68, 2588-2600 (1995).

[5] L. Song, C. A. Varma, J. W. Verhoeven, H.J. Tanke, Influence of the triplet excited state on the photobleaching kinetics of fluorescein in microscopy, Biophysical journal, 70, 2959-2968 (1996).

Supplementary 5: Tackling Optical Aberration in OVSS Imaging

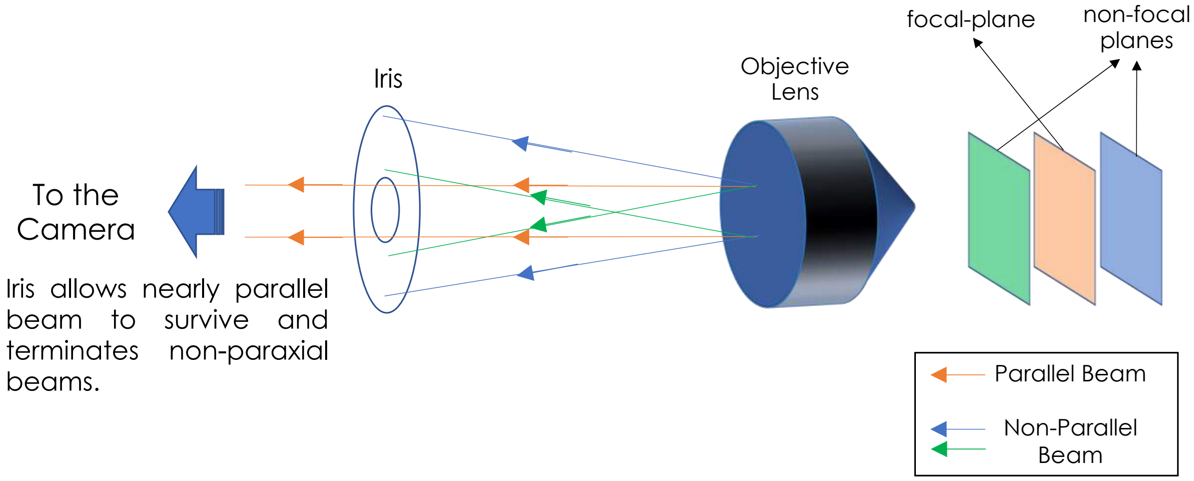

Fig. S8: Optical configuration that use Iris in the detection arm which lead to the reduction of optical aberration while data collection.

It may be noted that, most of the existing light-sheet microscopes employ water-dipping objectives. Although this reduces optical aberration, it makes the optical system somewhat limited and accessible. There are application that benefit from air-immersion objectives [1,2]. For our OVSS system, we wanted to avoid water-dipping lenses simply because this limits the use of light-sheet microscopy for a wide range of applications and makes the technique cumbersome. To take care of optical aberration, we have added IRIS in the detection-path to cut-off the non-paraxial rays. This substantially reduces optical aberration and ensures that most of the fluorescence entering the tube-lens (in the detector arm) are paraxial in nature.

This can as well be observed from the bead images obtained using OVSS system. Fig. S9 shows, (A) Fluorescent bead imaging with Iris-Open, and (B) Iris Closed. The reduction of optical aberration is quite evident. See supplementary videos 1 and 2.

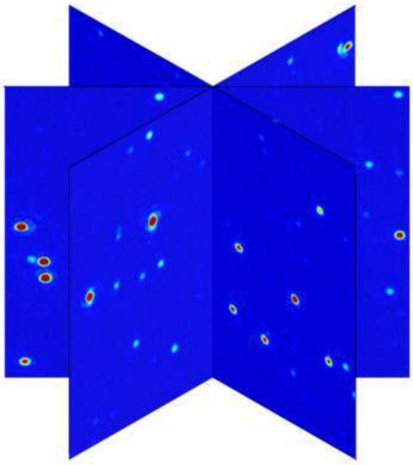

2D Angular Image obtained with Iris closed

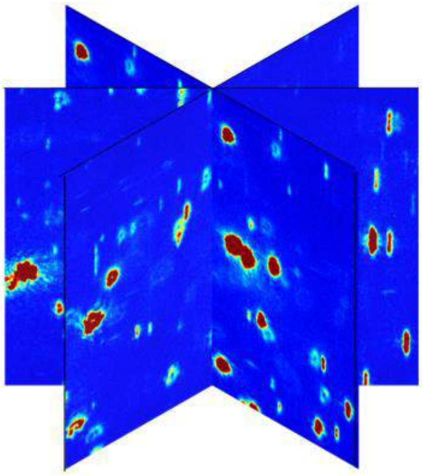

2D Angular Image obtained with Iris open

Fig. S9: 2D angular images of fluorescent beads with, (A) Iris Closed and (B) Iris open. The optical aberration is quite evident.

Caption for Supplementary Videos:

Supplementary Videos 1: Raw data of gel-embedded fluorescent beads without iris in the detection path.

Supplementary Videos 2: Raw images of gel-embedded fluorescent beads with iris in the detection path.

References:

[1] Chelur K. Rasmi, Sreedevi Padmanabhan, Kalyanee Shirlekar, Kanhirodan Rajan, Ravi Manjithaya, Varsha Singh, and Partha Pratim Mondal, Integrated light-sheet imaging and flowbased enquiry (iLIFE) system for 3D in-vivo imaging of multicellular organism, Appl. Phys. Lett. 111, 243702 (2017).

[2] Kavya Mohan, Partha Pratim Mondal, Light-sheet based lithography technique for patterning an array of microfluidic channels, Microscopy Research and Technique, 936-940, 81(9) 2018.

Supplementary 6: High Resolution Raw Images and Volume-Stack Using 10X/0.3NA Detection Objective in OVSS System

In zebrafish, from 16 hours post fertilization (h.p.f.), macrophages are produced by haematopoietic cells that initiate innate immunity [1][2][3]. In Zebrafish embryo, macrophases play important roles in development that include, tissue homeostasis and wound healing [4]. Macrophases are known to migrate throughout the body to carry out tasks such as tissue remodelling and pathologival processes. Here, we labelled macrophases with Bodipy dye (ex/em : 493 nm / 503 nm) that are located in Yolksac of Zebrafish embryo [5]. Fig.S10 shows accumulated microphases in the Yolksac of Zebrafis embryo during Segmentation period. Few random raw images (from a stack of 60 images required for volume reconstruction) obtained using OVSS imaging system are shown. The effect of bleaching and loss of fluorescence is quite evident that caused low signal-to-noise ratio. Severe photobleaching is observed during imaging.

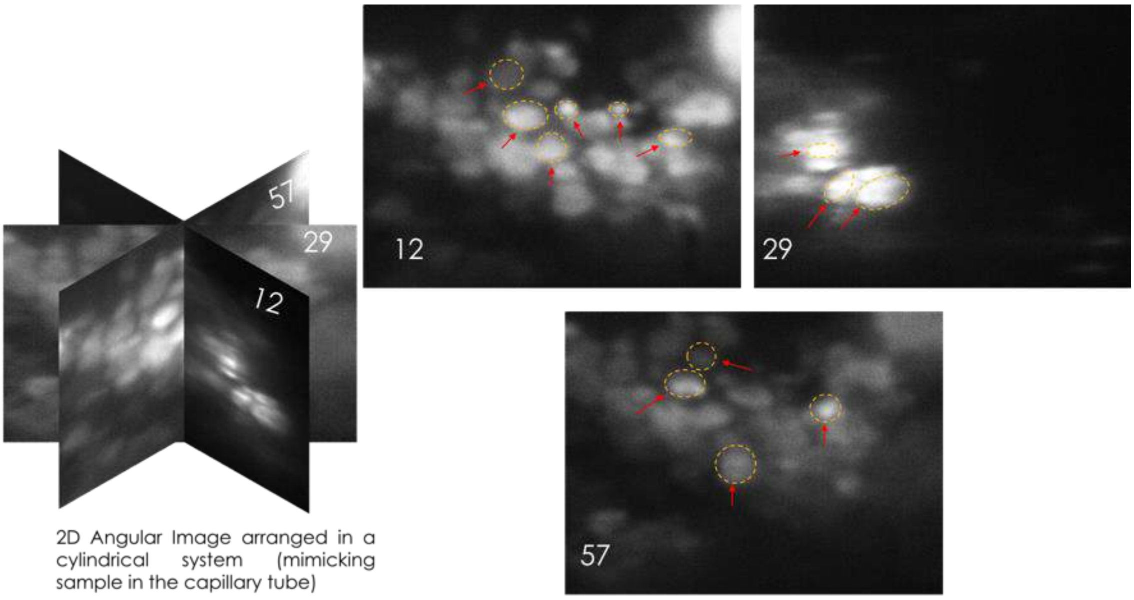

Fig.S10: High resolution images (planes, 12, 29 and 57 from raw volume stack) obtained using a 10X/0.3NA objective in the detection path of a OVSS imaging system. Circles and arrows show the presence of primitive macrophages 24 hours post fertilization.

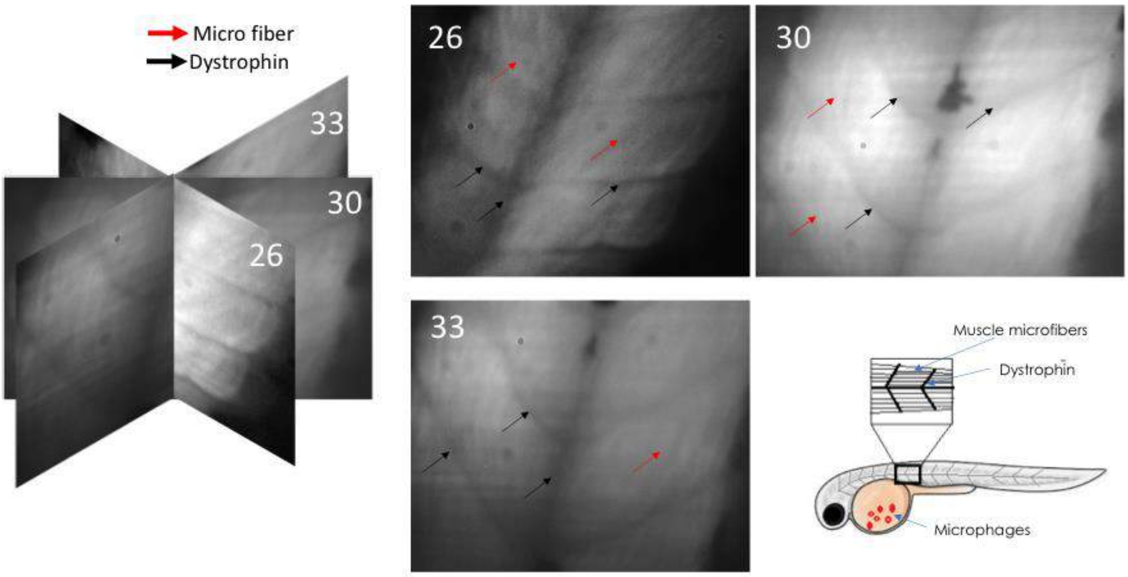

Fig.S11: High resolution images (planes, 26, 30 and 33 from raw volume stack) of muscle structure 24 hours post fertilization using a 10X/0.3NA detection objective. Red and black arrow indicates microfibre and Dystrophin, tespectively.

In addition, we have also imaged Zebrafish muscle. The muscle integrity is maintained by Dystrophin (Dmd) which is a structural protein that links the extracellular matrix to actin filaments in muscle microfibers. For visualization, we have used TRITC that binds to actin filaments. Fig.S11 shown the OVSS reconstructed images of zebrafish muscle detailing its fine structure that consists of Dmd and microfibers.

References:

[1] Guyader, D. L. e. et al. Origins and unconventional behavior of neutrophils in developing zebrafish origins and unconventional behavior of neutrophils in developing zebrafish. Blood 111, 132–141 (2008).

[2] Herbomel, P., Thisse, B. & Thisse, C. Ontogeny and behaviour of early macrophages in the zebrafish embryo. Development 126, 3735–3745 (1999)

[3] Primitive macrophages control HSPC mobilization and definitive haematopoiesis Jana Travnickova, Vanessa Tran Chau, Emmanuelle Julien, Julio Mateos-Langerak, Catherine Gonzalez, Etienne Lelièvre, Georges Lutfalla, Manuela Tavian & Karima Kissa Nature Communications volume 6, Article number: 6227 (2015).

[4] Wynn, T. a., Chawla, A. & Pollard, J. W. Macrophage biology in development, homeostasis and disease. Nature 496, 445–455 (2013).

[5] Knight M, Braverman J, Asfaha K, Gronert K, Stanley S (2018) Lipid droplet formation in Mycobacterium tuberculosis infected macrophages requires IFN-γ/HIF-1α signaling and supports host defense. PLoS Pathog 14(1): e1006874.

[6] Goody M F, Kelly M W, Reynolds C J, Khalil A, Crawford B D, C A Henry, NAD+ Biosynthesis Ameliorates a Zebrafish Model of Muscular Dystrophy. PLoS Biol 10, e1001409 (2012).

Supplementary 7: Shannon's Entropy : Measure of Information Content

To measure the information content of full volume image acquired by OVSS and traditional LSM, we employ Shannon's entropy [1] [2]. By definition, Entropy is a measure of image information content, which is interpreted as the average uncertainty of information source. It is frequently used for the quantitative analysis and evaluation of image details [1] [2]. Entropy for an image (with intensity / grey-level ***i***) is define as,

$$E = -\sum_{i} P_i \, log_2(P_i)$$

where P_i represents the corresponding histogram count.

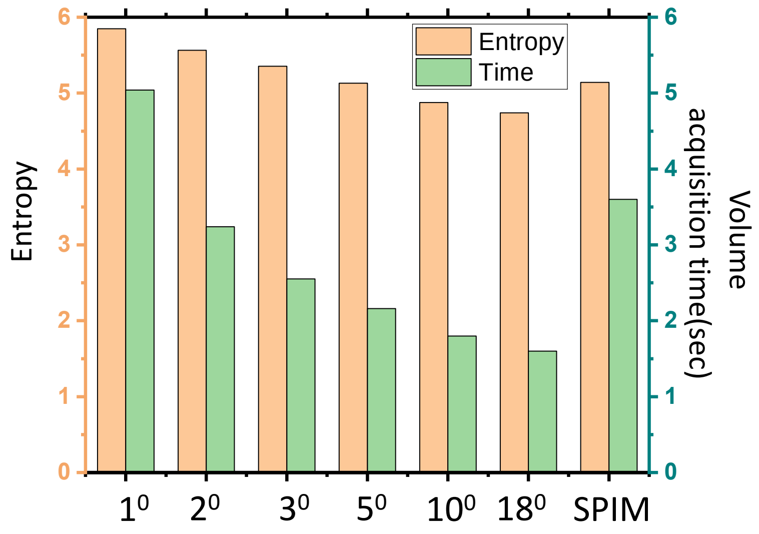

Fig. S12: Information content measure and speed: Bar-graph for OVSS system and its comparison with traditional LSFM (SPIM) system.

Fig. S12 shows the information content and speed (in terms of volume acquisition time) for OVSS and LSFM (SPIM) for the volume reconstructed stack of a Tm1 GFP labelled L3 stage *Droshophila* larvae. For a given volume section of specimen, OVSS (with 3 and 5 degree sampling) performs similar to standard LSM-SPIM system. This confirms that standard LSFM-SPIM (entropy value = 5.5) is comparable to OVSS (entropy value of 5.4 and 5.6 respectively for 5° and 3° , respectively.). In addition, we observed a trade-off between speed (volume acquisition time) and information content. Accordingly, we choose to work with a sampling of 3°. We noted that, acquiring angular data at higher sampling angle (θ) can even fasten the process, however angular data sampling at higher sampling rate reduces the entropy value resulting

in image quality degradation. So an optimal sampling angle needs to be used for OVSS imaging.

References:

[1] Information Entropy Measure for Evaluation of Image Quality Du-Yih Tsai,corresponding author1 Yongbum Lee,1 and Eri Matsuyama, J Digit Imaging. 2008 Sep; 21(3): 338–347.

[2] J. Lin, Divergence measures based on the Shannon entropy, IEEE Transactions on Information Theory, 37, 1 (2006).

Supplementary 8: Drosophila Larvae Imaging

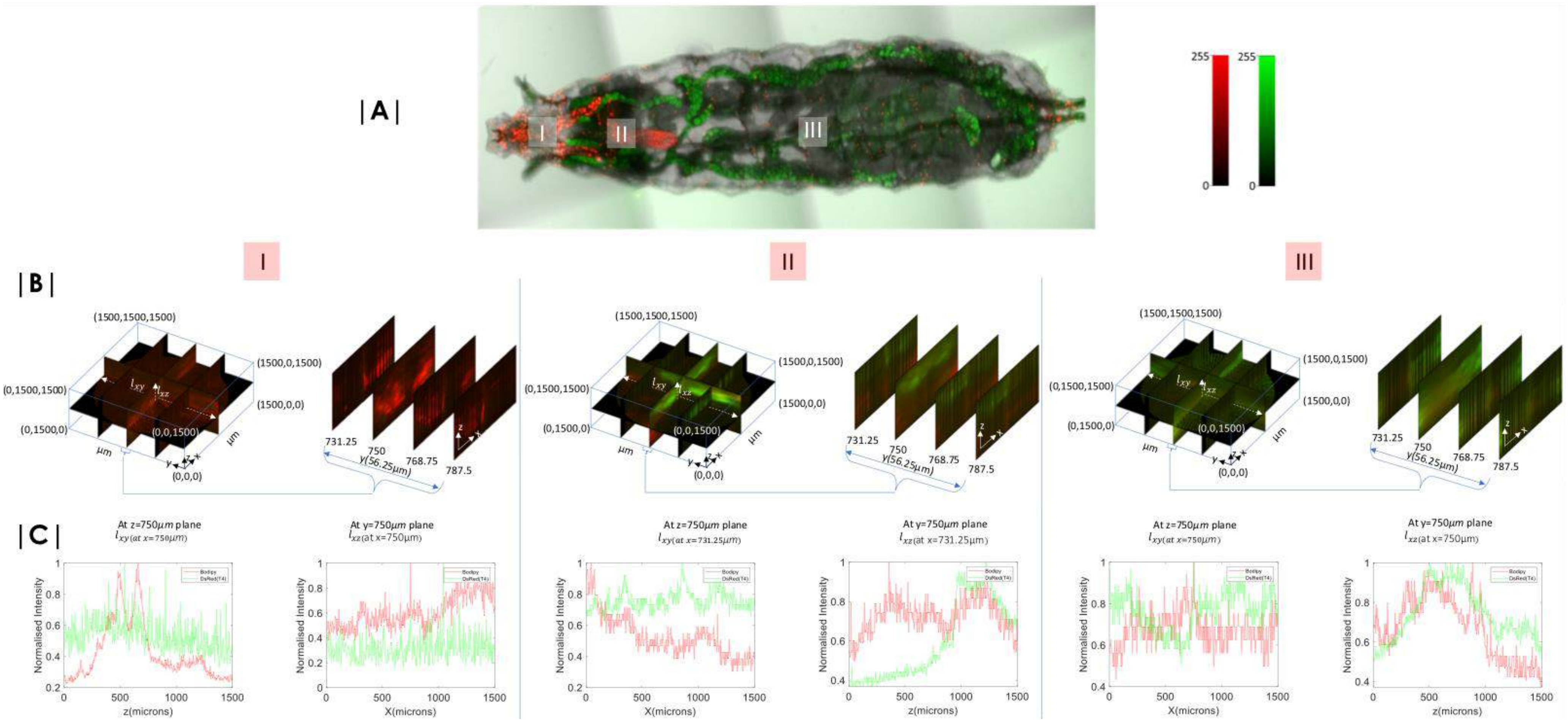

Fig. S13. *Drosophila* larvae imaging. (A) Leica confocal (equipped with a 10X objective) image of *Drosophila* larvae at L3 stage (taken at Bioimaging facilities, IISc). (B) OVSS reconstructed images of Drosophila larvae with nervous system expressed DsRed protein and fat body was labelled with BODIPY. Fat distribution is visible through chitinous covering spreading throughout the body following clustered globule thread like pattern. Nerves are clearly visible near the head region. Section I, II and III show different regions of *Drosophila* larva. (C) Line intensity shows the dominance of DsRed(T4) indicating nervous system in region I, whereas region II and II are predominantly fat bodies.

We have also demonstrated the two-color OVSS for imaging *Drosophila* larvae. In general, fine dissection is required to visualize internal tissues in a *Drosophila* larva because of its chitinous covering. Fig. S13 shows OVSS images and reconstructed volume stacks of an intact *Drosophila* third instar larva. We chose to simultaneously visualize two different tissue systems, i.e., the neurons and the fat body, in particular, because they are important and significant in the context of various pathological conditions such as Motor Neuron Diseases (MND) [1]. DsRed is expressed specifically in the neurons, and fat globules are labelled with BODIPY. Three different regions are shown in Fig. S13. In the first cross-section we have shown the volume image of the oral region, where DsRred is predominant over BODIPY, since the chemosensory nerves are prominent due to the sparse fat bodies. The second cross-section, posterior to the first, reveals the larval brain surrounded by fat bodies. The third cross-section shows volume images of abundant fat bodies, and DsRed-expressing neurons scattered throughout the abdominal region. This highlights the potential of OVSS for non-invasively imaging partly-transparent organisms.

Preparation of Fluorescent-labeled Drosophila Larva

All fly stocks were maintained on standard corn meal/ yeast/ sugar/ agar medium at 25° C. The pan-neuronal driver line, *Elav-Gal4* (Chromosome X), drives expression of GAL4 at all developmental stages [2]. *UAS-RedStinger* (Chromosome 3) was obtained from the Bloomington *Drosophila* Stock Center, Indiana. This line expresses, under the control of *UAS*, a variant DsRed protein tagged with the nuclear localization signal from the *Drosophila melanogaster tra* gene. DsRed(T4) is a tetrameric red fluorescent protein with an excitation peak of 555nm, and an emission peak of 586nm in vitro. This synthetic construct is a fast-maturing derivative of the naturally-occurring red fluorescent protein encoded by the *drFP583* gene of the coral *Discosoma* [3]. We labeled neurons with DsRed(T4) fluorophore and the fat body monitoring was achieved by staining it with BODIPY dye.

For multicolour staining of *Drosophila*, two distinct tissue systems were selected which are relevant to developmental studies. We chose fat body and neurons. Neurons were labelled with DS Red which is red fluorescent protein. And lipids were stained with BODIPY 493/503 (Invitrogen- D3922). BODIPY was diluted in PBS or DMSO and stock concentration of 2 mg/mL prepared. The stock DMSO solution is diluted 1:300 into

DMEM. For staining fat body, *Drosophila* larvae were first fixed in 3.7 % formaldehyde followed by PBS wash. Samples were then stained with 1:300 BODIPY 493/503 and incubated for 3 hours. After this, the larvae were washed with PBS and encaged in the gel-matrix filled capillary tube (Z114995 Sigma Aldrich). Prior to this, the capillary tube was first filled with filtered liquefied 1 % agarose gel. With the help of pipette larva sample was gently placed inside the capillary tube.

References:

[1] G. Pennetta and M. A. Welte, Emerging Links between Lipid Droplets and Motor Neuron Diseases. Developmental cell, 45(4), 427–432(2018).

[2] K. Khairy, W. C. Lemon, F. Amat and P. J. Keller, Light sheet-based imaging and analysis of early embryogenesis in the fruit fly, Methods Mol Biol., 1189, 79-97(2015).

[3] M. Pende, K. Becker, M. Wanis, S. Saghafi, R. Kaur, C. Hahn, N. Pende, M. Foroughipour, T. Hummel and H.-U. Dodt, High-resolution ultramicroscopy of the developing and adult nervous system in optically cleared yolk-sac melanogaster, Nature Communication, 9, 4731 (2018).

Supplementary 9: Time-Lapse Imaging

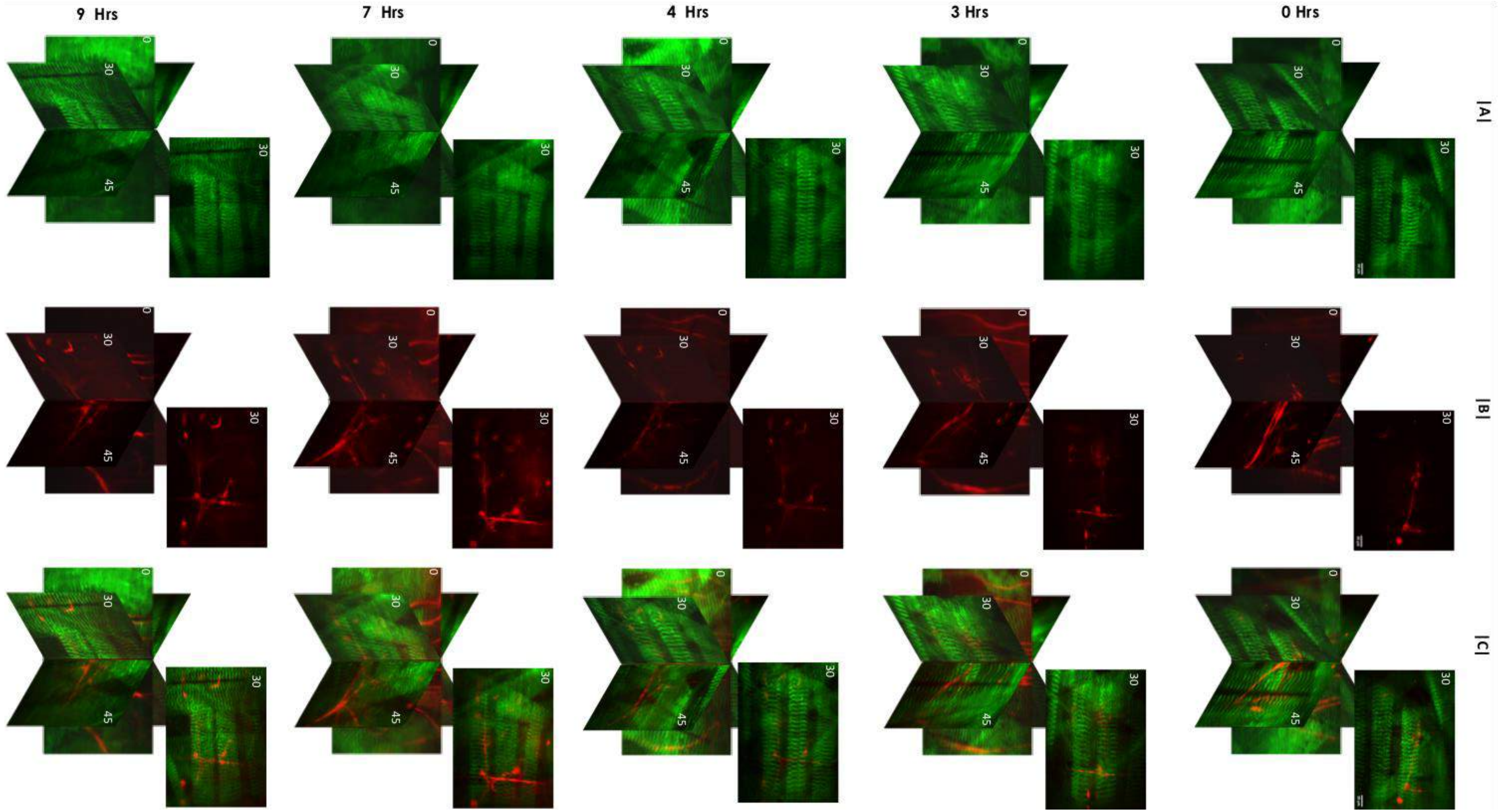

Fig. S14. Time lapse imaging of *Drosophila* larvae for 10 hrs. imaging. Images capture with 10x objective (0.25NA, Olympus) and focused with 200mm tube lens on sCMOS(Andor Zyla 4.2, 6.5 pixel size). Each frame of size 2048X2048 pixels image is captured with an exposure time of 30ms. Total 300 photons per assumed to be background noise while recording data. Dat. File recorded by sCOMS is converted into .tiff format using batch conversion of Solis. Two-colour transgenic L3 stage *drosophila* larvae screen volume angular 2D images was captured in every 3degree quantised step rotational in time lapse manner after every 2 minutes. Figure show the each channel volume image and corresponding merge volume image at time point 0hr, 3hrs,4hrs,7hrs and 9hrs. **(A)** Green channel: Volume stack image of muscle myofibril structure,**(B)** Red channel: Volume image of Neurons **(C)** Merged image show the co-development of muscle and neurons of L3 stage larva.

Time-lapse imaging of Drosophila (L3 stage larva) is carried out using OVSS light sheet system. A relay control chopper is used just after the dichroic beam-combiner in the illumination system. Chopper ON / OFF time is synchronised via Arduino in such a way that beam is allowed to illuminate the specimen only during data recording. This substantially reduces unnecessary exposure of specimen thereby minimising photobleaching. At each time point a total 120 2D planes are collected with 3 degree angular sampling. First 60 2D angular images are enough to reconstruct volume stack. Time lapse imaging up to 10 hrs is done. Figure shows that despite the optically dense structure of the Drosophila larva, both muscle and neuron features are well resolve. OVSS system shows the emergence of new feature and development of both muscle and neurons. This is evident from the widening of neurons at 9 hrs. High resolution imaging is further facilitated by the sCMOS (Andor Zyla 4.2) camera that has high quantum efficiency and efficient signal-to-noise ratio.
